# Automated Assay of a Four-Protein Biomarker Panel for Improved Detection of Ovarian Cancer

**DOI:** 10.3390/cancers13020325

**Published:** 2021-01-17

**Authors:** Christopher Walker, Tuan-Minh Nguyen, Shlomit Jessel, Ayesha B. Alvero, Dan-Arin Silasi, Thomas Rutherford, Sorin Draghici, Gil Mor

**Affiliations:** 1 Department of Obstetrics and Gynecology, Wayne State University, Detroit, MI 48201, USA; ck4145@wayne.edu (C.W.); Ayesha.alvero@wayne.edu (A.B.A.); 2 Department of Computer Science, Wayne State University, Detroit, MI 48201, USA; tuan.minh.nguyen@wayne.edu (T.-M.N.); sorin@wayne.edu (S.D.); 3 Department of Obstetrics, Gynecology & Reproductive Sciences, Yale University School of Medicine, New Haven, CT 06510, USA; shlomit.jessel@yale.edu (S.J.); dan-arin.silasi@mercy.net (D.-A.S.); 4 C.S. Mott Center for Human Growth and Development, Wayne State University, Detroit, MI 48201, USA; 5 Department of Obstetrics and Gynecology, University of South Florida, Tampa, FL 33606, USA

**Keywords:** ovarian cancer, early detection, CA-125, MIF, osteopontin, prolactin

## Abstract

**Simple Summary:**

The survival of patients diagnosed with ovarian cancer depends largely on the extent of the disease upon diagnosis. When confined to the ovaries, patients’ 10-year survival is more than 70%. This drastically drops to less than 5% when patients are diagnosed with far-advanced disease. Unfortunately, more than 80% of patients are diagnosed at advanced stage due to the lack of test for early detection. We report the development of a blood test measuring four proteins (macrophage migration inhibitory factor, osteopontin, prolactin and cancer antigen 125), which can distinguish ovarian cancer samples, even early-stage disease, from healthy samples in the population tested. This study is another step towards the application of a useful test for early detection of ovarian cancer that is both highly accurate and specific.

**Abstract:**

*Background*: Mortality from ovarian cancer remains high due to the lack of methods for early detection. The difficulty lies in the low prevalence of the disease necessitating a significantly high specificity and positive-predictive value (PPV) to avoid unneeded and invasive intervention. Currently, cancer antigen- 125 (CA-125) is the most commonly used biomarker for the early detection of ovarian cancer. In this study we determine the value of combining macrophage migration inhibitory factor (MIF), osteopontin (OPN), and prolactin (PROL) with CA-125 in the detection of ovarian cancer serum samples from healthy controls. *Materials and Methods*: A total of 432 serum samples were included in this study. 153 samples were from ovarian cancer patients and 279 samples were from age-matched healthy controls. The four proteins were quantified using a fully automated, multi-analyte immunoassay. The serum samples were divided into training and testing datasets and analyzed using four classification models to calculate accuracy, sensitivity, specificity, PPV, negative predictive value (NPV), and area under the receiver operating characteristic curve (AUC). *Results*: The four-protein biomarker panel yielded an average accuracy of 91% compared to 85% using CA-125 alone across four classification models (*p* = 3.224 × 10^−9^). Further, in our cohort, the four-protein biomarker panel demonstrated a higher sensitivity (median of 76%), specificity (median of 98%), PPV (median of 91.5%), and NPV (median of 92%), compared to CA-125 alone. The performance of the four-protein biomarker remained better than CA-125 alone even in experiments comparing early stage (Stage I and Stage II) ovarian cancer to healthy controls. *Conclusions*: Combining MIF, OPN, PROL, and CA-125 can better differentiate ovarian cancer from healthy controls compared to CA-125 alone.

## 1. Introduction

Ovarian cancer remains the leading cause of death from gynecologic cancers [1]. Although the 5-year survival rate of ovarian cancer can be as high as 90%, this is observed in only 20% of patients – those who are diagnosed at an early stage (Stage I or Stage II) [1,2]. Unfortunately, most patients are diagnosed at advanced stage (Stage III and Stage IV), with a 5-year survival rate of approximately 30% [2]. Early symptoms of ovarian cancer are nonspecific and the lack of a cost-effective method to detect early stage disease results in 80% of diagnoses at an advanced stage [2,3]. Ovarian cancer screening continues to be a challenging and elusive undertaking. 

The difficulty in achieving a good screening test for ovarian cancer lies in the need for both a high sensitivity and specificity due to the low prevalence of the disease [4]. A screening test should be able to identify those who require further investigation and diagnostic intervention. Treatment of ovarian cancer universally involves invasive surgery and chemotherapy. Thus, an ovarian cancer screening test should have a low number of false positives to reduce unnecessary surgery and associated morbidity. For a disease such as ovarian cancer, with an inicudence of 40 per 100,000 per year, the recommended cut offs for an acceptable screening test is sensitivity of greater than 75% and a high specificity, preferably greater than 99.6%, in order to obtain a high positive predictive value (PPV) of 10% [4,5]. 

Multiple screening and detection methods have been evaluated [6]. The most historically recognized test is cancer antigen-125 (CA-125), discovered by Bast and colleagues in 1981 [7]. Although a clinically useful biomarker, serum CA-125 is a poor screening test due to its relatively low sensitivity, specificity and positive predictive value [8]. Other screening strategies currently being investigated use the combination of CA-125, transvaginal ultrasound and multi-step algorithms, which demonstrate higher sensitivities and specificities compared to CA-125 alone. These methods however require appropriate facilities and experienced ultrasonographers. Thus, although utilizing multi-step algorithms are showing improved statistical success, biomarker investigations should not be abandoned due to the efficiency, convenience, cost-effectiveness and increased compliance to a blood test [8,9,10]. 

We have previously described and investigated a panel of six serologic protein biomarkers (leptin, prolactin (PROL), osteopontin (OPN), insulin-like growth factor II (IGF-II), macrophage migration inhibitory factor (MIF), and CA-125) for the detection of ovarian cancer with a sensitivity of 95.3% and specificity of 99.4% [11,12]. Individual analysis of all six biomarkers however, showed that leptin and IGF-II had the lowest sensitivity and specificity. Thus, the objective of this study is to assess the performance of the four top-performing markers, MIF, OPN, PROL and CA-125 in detecting ovarian cancer patients from healthy controls in a retrospective study comprised of patients with both early and late-stage ovarian cancer. More recently, Guo et al. validated two of these biomarkers and demonstrated that the combination OPN, MIF, CA-125 and anti-IL-8 autoantibodies was able to increase detection of ovarian cancer compared to CA125 alone [13]. 

To quantify MIF, OPN, PROL and CA-125, we used the SimplePlex^TM^ platform, a fully automated microfluidic immunoassay, which uses an automated analyzer, Ella, and a multi-analyte specific cartridge. The benefits of SimplePlex^TM^ include the fact that it is fully automated, validated, rapid and reproducible with high sensitivity, when compared to conventional ELISAs [14]. 

This protein biomarker panel was used for each type of classifier, in two patient cohorts, one used for training and the other used for validation. We used four different classification models: KNN, logistic regression, random forest and support vector machines (SVM). Our results show that MIF, OPN and PROL, in combination with CA-125 provide statistically significant higher sensitivity, specificity and accuracy when compared to CA-125 alone. 

## 2. Results

### 2.1. Development of the Test Model

Our objective is to determine the combined utility of CA-125, MIF, OPN and PROL in distinguishing ovarian cancer serum samples from healthy controls and compare its usefulness to CA-125 alone as a single biomarker. We used serum samples from a total of 153 patients diagnosed with ovarian cancer and 279 serum samples from age-matched healthy controls. We first divided the samples into two cohorts, cohort 1 and cohort 2 (Table 1) for the purpose of using them as training and testing sets. Within each cohort, samples were subdivided into cancer (C) and healthy (H) datasets. The cancer samples were further subdivided into early stage (E) and late stage datasets (L) (Table 1). Each data set (e.g., E1 or early-stage cancer cohort 1) was interchangeably assigned to training and testing groups (Table 2) to produce the most representative, reproducible and unbiased data. At all times, we trained our models on one data set and test on a completely different set. First, we used E1 dataset (serum samples from early-stage ovarian cancer patients from cohort 1 versus H1 dataset (serum samples from healthy controls from cohort 1 to both train and test the model (Experiment ID#1 in Table 2, Table 3, Table 4, Table 5 and Table 6). This was followed by testing 17 other experiments using different dataset combinations as shown in Table 2, Table 3, Table 4, Table 5 and Table 6. We used KNN, logistic regression, random forest, and SVM, which are four commonly used classification models, to analyze the statistical success of using combination CA-125, MIF, OPN, and PROL versus using CA-125 alone. The complete reports of all statistical measures, namely accuracy, sensitivity, specificity, PPV, NPV and AUC are shown in Table 2, Table 3, Table 4, Table 5, Table 6 and Table 7 respectively. The PPV and NPV were calculated on the indicated cohorts and do not represent the PPV and NPV expected of this panel in the general population. This is because the OVCA will have a much lower prevalence in the general population compared with these cohorts. 

Table 2 shows the individual and average accuracies obtained from the four classification models. The average accuracy is shown for each of the 18 experiments and compares the average accuracy when the four-protein panel was used compared to CA-125 alone. The combined usage of CA-125, MIF, PROL, and OPN provides better average accuracies than the use of CA-125 alone in all experiments regardless of the dataset used to train and test the model. The only exception is experiment 16 (Table 2). Despite the lower accuracy however, the four-protein panel yields much higher sensitivities, demonstrating better ability to detect ovarian cancer from healthy controls compared to CA-125 alone (Table 3). 

The raw data, i.e., the contingency matrices and the ROC curves along with Youden index used to construct the Table 2, Table 3, Table 4, Table 5, Table 6 and Table 7, are provided in the Supplementary Materials (Tables S1–S72 and Figures S1–S18).

### 2.2. Accuracies of the Models

**Figure 1:**
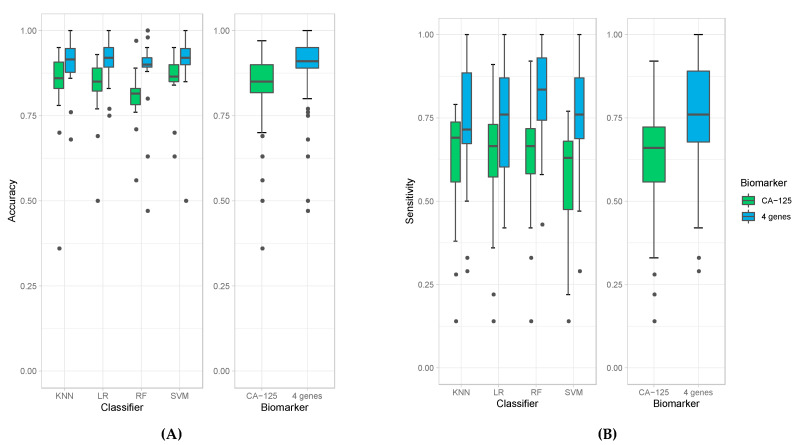

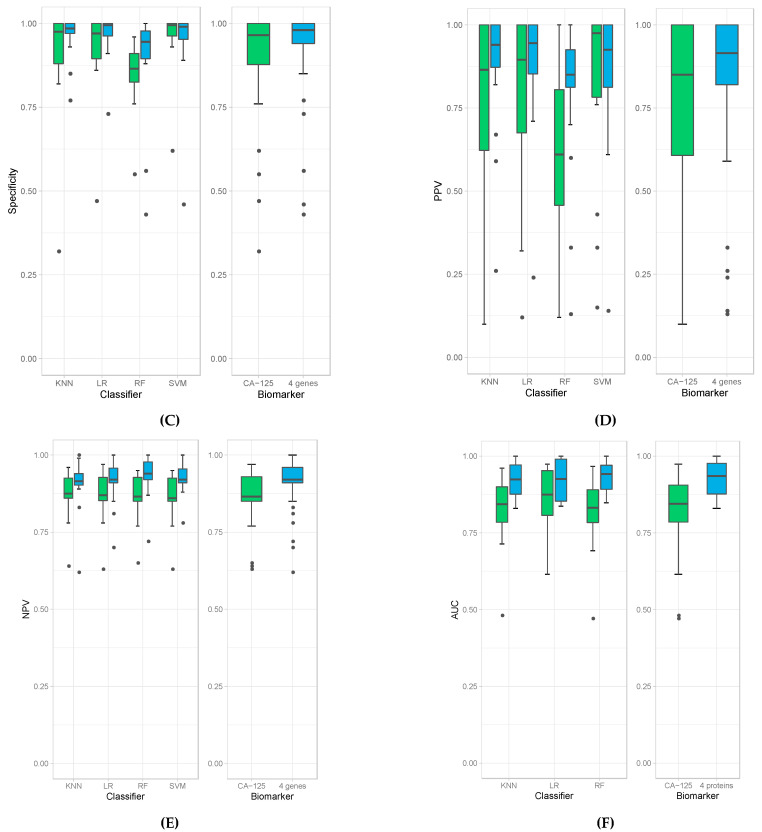
Box plots of accuracies (**A**), sensitivities (**B**), specificity (**C**), PPV (**D**), NPV (**E**), and AUC (**F**) obtained by individual classifiers (left panel), namely K-Nearest Neighbor (KNN), Logistic Regression (LR), Random Forest (RF), and Support Vector Machine (SVM), and average accuracies of all models (right panel) when using CA-125 alone (green bars) and using all four genes (blue bars).

We then evaluated the accuracies obtained from all 18 experiments for each of the four classification models (left panel) as well as average accuracies for all the models. When the four-protein panel was used, we observed a median accuracy of 91% compared to 85% median accuracy when CA-125 was used alone (Figure 1A). This demonstrates that the four-protein panel performed better than CA-125 alone in distinguishing cancer (irrespective of stage) from healthy controls and in distinguishing late-stage cancer from healthy controls. More importantly, the use of the four-protein panel also performed better than CA-125 alone in distinguishing early-stage ovarian cancer from healthy control. The Wilcoxon *p*-value = 3.224 × 10^−9^ indicates that this is superior and statistically significant. The four-protein panel also demonstrated better sensitivity (median of 76%), specificity (median of 98%), PPV (median of 91.5%), NPV (median of 92%) and AUC (median of 93.5%) compared to CA-125 alone in distinguishing ovarian cancer from healthy controls (Wilcoxon *p*-values: 2.22 × 10^−7^, 0.02, 1.27 × 10^−2^, and 3.629× 10^−7^and and 2.34 × 10^−7^, respectively; Figure 1B–F).

The results obtained from the four classification models used in these experiments are comparable but with some differences. All of them derive high accuracies in all experiments, especially when using the four-protein panel (e.g., all median accuracies are over 0.9). Among all classification models, random forest produced the highest sensitivity; however, it resulted in the lowest specificity (Figure 1B,C). Random forest models also produced the highest median value of NPV (Figure 1E) and AUC (Figure 1F). The highest specificity and PPV are achieved with logistic regression (Figure 1D). Due to these variations amongst classification models, we report the average calculations of the classification models to obtain the most unbiased, representative and reliable data in terms of comparing the four-protein panel versus CA-125 alone.

In the experiments that used samples from patients with early stage (Stage I/II) ovarian cancer for training, the sensitivities to detect cancer are notably lower than in experiments that used samples from late-stage patients (Stage III/IV) (Wilcoxon *p*-value = 7.78 × 10^−8^). This result is likely due to the lower quantity of samples of patients with early stage (*n* = 37) compared to late stage ovarian cancer (*n* = 116).

## 3. Discussion

In the current study, we investigated the ability of four serologic protein biomarkers to distinguish ovarian cancer serum samples from healthy control serum samples using an automated, multi-analyte, microfluidic cartridge-based immunoassay. Compared to conventional ELISA and other multiplex methods, the Simple Plex assay allows for more automated, reproducible, and rapid assays in triplicates using small volume samples [14]. The four-protein biomarker panel yielded an accuracy of 91% compared to 85% using CA-125 alone across all the classification models, which was statistically significant (*p* = 3.224 × 10^−9^). Further, the four-protein model produced a higher sensitivity (median of 76%), specificity (median of 98%), PPV (median of 91.5%), NPV (median of 92%) and and AUC (median of 93.5%) compared to CA-125. These PPV and NPV are those for the test samples used in this study. We do recognize that the high PPV values (> 90%) are due to the high prevalence of ovarian cancer in our cohort, compared to the general, or even a high-risk population. 

Ovarian cancer is the leading cause of mortality amongst gynecologic cancers and the fifth most common cause of death in women [1]. The problem lies with the inability to diagnose ovarian cancer at an early stage, when the prognosis is favorable. An effective method for early detection would most certainly help mitigate this burden. Thus, an ongoing goal amongst gynecologic oncology investigators is to develop methods that are both accurate in detecting early stage disease, but also one that limits false positives. The other advantage of this biomarker panel may be its use to detect early recurrence or persistence after treatment.

There is a general consensus that a serologic screening test for the general population is essentially futile, unless extremely high specificity is attained, due to the very low prevalence of ovarian cancer in the population [6]. However, in patients who are at high risk of ovarian cancer, such as patients with BRCA1/2 mutations, there may be some utility and benefit [15]. Serologic tests have the benefit of cost effectiveness, availability and are least invasive. The candidate protein biomarkers included in this study are CA-125, MIF, OPN, and PROL. Individually, they have demonstrated to be elevated in patients with ovarian cancer when compared to healthy individuals [7,16,17,18]; however, this exact combination was yet to be studied for the purpose of early detection.

CA-125 is a high-molecular-weight membrane glycoprotein recognized by the monoclonal antibody OC 125 [7]. It was originally described to be increased in the serum of at least 80% of patients with epithelial ovarian cancer [19]. Since its inception, CA-125 continues to play a large role in the diagnosis and surveillance of ovarian cancer. In regards to screening, although CA-125 is not adequate by itself, it continues to be studied and integrated into screening algorithms [5,20,21,22,23] resulting in higher sensitivities and specificities.

MIF is an inflammatory cytokine originally described to be a product of activated T lymphocytes and played a primary role in inhibiting macrophage migration [16,24]. However, monocytes and macrophages were later implicated as the primary site for the production of MIF, typically following exposure to various toxins, bacteria or cytokines [25,26,27]. MIF is a key regulator in various immune and inflammatory pathways and a by-product of many cells and tissues [24]. More importantly, MIF has been found to have high circulating levels in various malignancies, including epithelial ovarian cancer [28,29]. The mechanism likely involves its role in the inflammatory process, angiogenesis, p53 expression and apoptosis associated with malignant neoplasia [16,29]. 

Osteopontin (OPN) is a secreted calcium-binding glycophosphoprotein that is involved in a number of biological processes, including inflammation, angiogenesis, immunity and tumor development [30,31]. Osteopontin is known to be overly expressed in several malignant tissues, including ovarian cancer cells [17]. Furthermore, it has been associated with late disease progression and tumor metastasis [17,32]. The phenotypic effects of OPN are likely due to activation of the MAPK, NF-kB, and PI3-K/Akt pathways [33,34]. These discoveries led way for OPN as a biomarker candidate for the detection of ovarian cancer [35,36]. 

Prolactin is a hormone secreted predominantly by lactrotroph cells of the anterior pituitary gland. It is involved in numerous biological processes, most notably, lactation and reproduction [37]. Prolactin is also produced in many other tissues, including the ovaries [38]. Previous studies have revealed that prolactin leads to growth and migration of ovarian epithelial cells, as well as inhibition of apoptosis [39,40]. Prolactin has been demonstrated to be elevated in serum samples of patients with ovarian cancer compared to healthy individuals and thus a biomarker of interest for the early detection of ovarian cancer [18].

We sought to analyze these proteins to characterize their sensitivity, specificity, PPV, NPV and overall accuracy in patients with early and late stage ovarian cancer versus healthy. Since CA-125 has been a longstanding historical serum protein biomarker for monitoring patients’ cancer progression, we used this as a reference marker when comparing our four-protein model. We analyzed them using two separate cohort sets, which were performed in different laboratories, with different serum samples and different experimenters to prove reproducibility. Classification models used were k-nearest neighbors algorithm (KNN), logistic regression, random forest, and support vector machine (SVM). 

Averaging all results of the four classification models, we determined that the sensitivity, specificity, PPV, NPV, AUC and overall accuracy with our 4-protein model faired better than CA-125 alone. In the clinical setting, CA-125 values considered abnormal are typically above 35 U/mL in the postmenopausal patient [41]. One of the major issues with CA-125 is that it is nonspecific to ovarian cancer leading to high false positives, which results in healthy patients undergoing unnecessary surgical intervention [42]. Adding protein biomarkers to CA125 could improve detection of cancers otherwise not identified by CA125 alone. 

It should be acknowledged that the PPV and NPV reported are those for the tested samples in our relatively small cohort and not for the general population. Given that the incidence of ovarian cancer in the general population is low at 40 per 10,000 a year, the value of such diagnostic test will best fit the screening of high-risk population. The high-risk population in ovarian cancer are those with known genetic mutation such as BRCA1 and BRCA2 and those with known history of early-onset ovarian cancer in first degree relatives. Our group has an on-going longitudinal study to determine the value of the described four-biomarker panel in detecting ovarian cancer in the high-risk population. It should also be acknowledged that limitations of this study are the limited number of samples and the lack of serial samples from the same patient. 

This study is another step forward towards developing a useful serologic biomarker panel for early detection of ovarian cancer that is both highly accurate and specific. The value of these markers and its potential application is not based on a single test, but monitoring their changes through multiple tests. We would like to apply our models and the novel SimplePlex assay to a larger cohort of high-risk patients prospectively, including serial samples from the same patient to determine the fluctuations between normal ranges and the modifications associated with the presence of the disease. 

## 4. Materials and Methods 

### 4.1. Patient Population

The ovarian cancer group (*n* = 153, average age = 57.1 year) was composed of women with newly diagnosed ovarian cancer following discovery of pelvic mass. Serum samples were collected in the clinic prior to surgery. The diagnosis was made following histopathologic evaluation of the surgical specimen by a pathologist. Of the 153 ovarian cancer patients, 37 were diagnosed with stage I/II and 116 were diagnosed with stage III/IV. Table 8 shows the breakdown of stages and histological diagnosis. The healthy control group (*n* = 279) was composed of age-matched healthy individuals who presented for routine gynecologic examination and had no evidence of ovarian or other cancer. These women also had no known personal risk factors for development of ovarian cancer and were disease free at least 6 months after sample collection.

### 4.2. Sample Collection

Serum samples were obtained with informed consent and collected under the approval of Yale University School of Medicine Human Investigations Committee. Collection, preparation, and storage of the blood samples were done as previously described [11,12] using guidelines set by the National Cancer Institute Inter-group Specimen Banking Committee. 

### 4.3. Simple Plex^TM^ Immunoassay

Levels of CA-125, MIF, OPN and PROL were quantified using the fully automated immunoassay platform, Ella (Protein Simple/Biotechne, San Jose, CA, USA) as previously described [43]. All four proteins were quantified using a single disposable microfluidic SimplePlex^TM^ cartridge, which holds up to 32 serum samples per assay. The serum samples were thawed on ice and diluted 1:10 with manufacturer’s provided diluent. 50 uL of diluted serum were added to each well of the SimplePlex^TM^ cartridge and the cartridge was placed in Ella for automated analysis and quantitation. Within the cartridge, each serum sample gets further divided into four unique microfluidic parallel channels that are specific for each of the four proteins being analyzed. Each protein channel contains three analyte-specific glass nanoreactors, which allows for each serum samples to be run in triplicates for each of the four protein samples. The following raw data were obtained: mean relative fluorescence units (RFUs), relative fluorescence units as percentage of coefficient of variance (RFU% CV), mean concentration and concentration as percentage of coefficient of variance. The mean concentration was used to perform the statistical analysis.

### 4.4. Statistical Analysis

A total of 432 serum samples (153 serum samples from ovarian cancer patients and 279 serum samples from healthy controls) were evaluated in this study. From these serum samples, we created several different cohorts for the purpose of training and testing (Table 8). A first cohort included a total of 153 serum samples, with 80 samples from healthy controls and 73 samples from ovarian cancer patients. In this set, 18 samples were from patients diagnosed with Stage I/II ovarian cancer and 55 samples were from patients diagnose with Stage III/IV. A second cohort included a total of 279 serum samples, with 199 samples from healthy controls and 80 samples from ovarian cancer patients. In this set, 19 samples were from patients diagnosed with Stage I/II ovarian cancer and 61 samples were from patients diagnose with Stage III/IV. Four classification models KNN, logistic regression, Random forest, and SVM were first trained and then used to predict whether a sample was ovarian cancer or healthy. Subsequently, we calculated the panel’s accuracy, sensitivity, specificity, PPV, NPV and area under the receiver operating characteristic curve (AUC) in differentiating ovarian cancer serums from healthy controls. Notice since SVM does not derive a continuous class probability/score but a fixed classification threshold instead, the ROC is not useful in this case and hence we did not include AUC of SVM in our comparison. For each classifier, the performance indicators above were calculated using only test samples, not used during the training of that particular classifier.

## 5. Conclusions

Combining MIF, OPN, PROL, and CA-125 can better differentiate ovarian cancer from healthy controls compared to CA-125 alone. This study is another step towards developing a useful serologic biomarker panel for early detection of ovarian cancer that is both highly accurate and specific.

## Figures and Tables

**Table 1:** Breakdown of each sample population in each cohort.

Cohort	Disease Stage	*(n)*	Designation in Table 2
**Cohort 1**		153	
**Healthy control**		80	H1
**Ovarian cancer**		73	C1
	**Early stage**	18	E1
	**Late stage**	55	L1
**Cohort 2**		279	
**Healthy control**		199	H2
**Ovarian cancer**		80	C2
	**Early stage**	19	E2
	**Late stage**	61	L2

**Table 2:** Comparison of accuracy between CA-125 alone and all four proteins (CA-125, MIF, OPN, and PROL) when using the four classification methods, K-Nearest Neighbor (KNN), Logistic Regression (LR), Random Forest (RF), and Support Vector Machine (SVM), in various experiments. In each experiment, “training” column and “testing” column list the subsets used for training and testing the classification models. In these columns, the letters “C”, “E”, “L”, and “H” denote serum samples from ovarian patients, from patients in early stage, in late stage of ovarian cancer, and healthy samples, respectively. The suffix, e.g., 1 or 2, indicates that samples are from cohort set 1 and cohort set 2. The accuracy measures the ability to correctly label the testing samples and is defined as the ratio of the number of samples correctly classified to the total number of samples. We highlighted the cell(s) with best accuracy in each experiment. In general, using logistic regression and all four proteins yield the best results on most occasions.

ID	Training	Testing	CA-125					4 Proteins				
			KNN	LR	RF	SVM	Mean	KNN	LR	RF	SVM	Mean
1	E1 vs. H1	E1 vs. H1	0.95	0.77	0.97	0.95	0.91	0.97	1	0.92	0.95	0.96
2	E1 vs. H1	E2 vs. H2	0.86	0.85	0.80	0.90	0.85	0.97	0.96	0.95	0.94	0.96
3	E1 vs. H1	C2 vs. H2	0.85	0.85	0.83	0.85	0.85	0.86	0.83	0.88	0.89	0.87
4	L1 vs. H1	L1 vs. H1	0.91	0.93	0.89	0.91	0.91	1	1	1	1	1.00
5	L1 vs. H1	L2 vs. H2	0.83	0.83	0.83	0.89	0.85	0.91	0.92	0.90	0.91	0.91
6	L1 vs. H1	C2 vs. H2	0.78	0.82	0.78	0.85	0.81	0.90	0.91	0.90	0.90	0.90
7	C1 vs. H1	E2 vs. H2	0.36	0.50	0.56	0.63	0.51	0.76	0.75	0.47	0.50	0.62
8	C1 vs. H1	L2 vs. H2	0.83	0.85	0.81	0.87	0.84	0.90	0.89	0.90	0.89	0.90
9	C1 vs. H1	C2 vs. H2	0.80	0.82	0.76	0.85	0.81	0.87	0.89	0.89	0.90	0.89
10	E2 vs. H2	E2 vs. H2	0.91	0.91	0.87	0.91	0.90	0.93	0.95	0.93	0.93	0.94
11	E2 vs. H2	E1 vs. H1	0.86	0.86	0.84	0.86	0.86	0.94	0.97	0.98	0.97	0.97
12	E2 vs. H2	C1 vs. H1	0.70	0.69	0.71	0.70	0.70	0.68	0.77	0.80	0.85	0.78
13	L2 vs. H2	L2 vs. H2	0.87	0.85	0.81	0.85	0.85	0.91	0.90	0.90	0.91	0.91
14	L2 vs. H2	L1 vs. H1	0.91	0.90	0.82	0.90	0.88	0.95	0.95	0.91	0.95	0.94
15	L2 vs. H2	C1 vs. H1	0.87	0.86	0.79	0.85	0.84	0.92	0.91	0.90	0.93	0.92
16	C2 vs. H2	E1 vs. H1	0.92	0.92	0.76	0.92	0.88	0.87	0.92	0.63	0.90	0.83
17	C2 vs. H2	L1 vs. H1	0.90	0.90	0.83	0.90	0.88	0.93	0.95	0.92	0.95	0.94
18	C2 vs. H2	C1 vs. H1	0.86	0.86	0.82	0.84	0.85	0.95	0.95	0.90	0.94	0.94

**Table 3:** The comparison of sensitivity between CA-125 alone and all four proteins (CA-125, MIF, OPN, and PROL) when using the four classification techniques. The sensitivity measures the ability to correctly identify condition samples and is defined as the ratio of the number of condition samples correctly classified to the total number of condition samples. We highlighted the cell(s) with best sensitivity in each experiment. Using random forest and all four proteins yield the best results on most occasions.

ID	Training	Testing	CA-125					4 Proteins				
			KNN	LR	RF	SVM	Mean	KNN	LR	RF	SVM	Mean
1	E1 vs. H1	E1 vs. H1	0.71	0.86	0.71	0.71	0.75	0.86	0.57	1	0.71	0.79
2	E1 vs. H1	E2 vs. H2	0.58	0.63	0.58	0.47	0.57	0.68	0.58	0.74	0.47	0.62
3	E1 vs. H1	C2 vs. H2	0.55	0.56	0.59	0.49	0.55	0.50	0.42	0.63	0.66	0.55
4	L1 vs. H1	L1 vs. H1	0.77	0.91	0.92	0.77	0.84	1	1	1	1	1.00
5	L1 vs. H1	L2 vs. H2	0.66	0.66	0.66	0.62	0.65	0.69	0.74	0.75	0.74	0.73
6	L1 vs. H1	C2 vs. H2	0.66	0.65	0.65	0.59	0.64	0.70	0.76	0.78	0.76	0.75
7	C1 vs. H1	E2 vs. H2	0.79	0.74	0.63	0.68	0.71	0.84	0.89	0.89	0.89	0.88
8	C1 vs. H1	L2 vs. H2	0.72	0.67	0.67	0.64	0.68	0.69	0.74	0.75	0.74	0.73
9	C1 vs. H1	C2 vs. H2	0.74	0.70	0.69	0.64	0.69	0.73	0.76	0.76	0.76	0.75
10	E2 vs. H2	E2 vs. H2	0.14	0.14	0.14	0.14	0.14	0.29	0.57	0.43	0.29	0.40
11	E2 vs. H2	E1 vs. H1	0.28	0.22	0.33	0.22	0.26	0.67	0.83	0.89	0.83	0.81
12	E2 vs. H2	C1 vs. H1	0.38	0.36	0.42	0.37	0.38	0.33	0.52	0.58	0.68	0.53
13	L2 vs. H2	L2 vs. H2	0.54	0.46	0.58	0.46	0.51	0.67	0.67	0.71	0.67	0.68
14	L2 vs. H2	L1 vs. H1	0.78	0.78	0.76	0.76	0.77	0.89	0.87	0.95	0.87	0.90
15	L2 vs. H2	C1 vs. H1	0.73	0.70	0.71	0.68	0.71	0.89	0.81	0.93	0.86	0.87
16	C2 vs. H2	E1 vs. H1	0.67	0.61	0.72	0.56	0.64	0.94	0.94	0.94	0.94	0.94
17	C2 vs. H2	L1 vs. H1	0.76	0.76	0.78	0.75	0.76	0.87	0.87	0.93	0.87	0.89
18	C2 vs. H2	C1 vs. H1	0.71	0.70	0.77	0.67	0.71	0.89	0.90	0.92	0.88	0.90

**Table 4:** The comparison of specificity between CA-125 alone and all four proteins (CA-125, MIF, OPN, and PROL) when using the four classification techniques, namely K-Nearest Neighbor (KNN), Logistic Regression (LR), Random Forest (RF), and Support Vector Machine (SVM). The specificity measures the ability to correctly identify control samples and is defined as the ratio of the number of control samples correctly classified to the total number of control samples. We highlighted the cell(s) with best specificity in each experiment. Using logistic regression and all four proteins yield the best results on most occasions.

ID	Training	Testing	CA-125					4 Proteins				
			KNN	LR	RF	SVM	Mean	KNN	LR	RF	SVM	Mean
1	E1 vs. H1	E1 vs. H1	1	1	0.78	1	0.95	1	1	1	1	1.00
2	E1 vs. H1	E2 vs. H2	0.88	0.87	0.82	0.94	0.88	0.99	0.99	0.97	0.95	0.98
3	E1 vs. H1	C2 vs. H2	0.97	0.97	0.92	0.99	0.96	1	1	0.98	0.97	0.99
4	L1 vs. H1	L1 vs. H1	1	0.94	0.94	1	0.97	1	1	1	1	1.00
5	L1 vs. H1	L2 vs. H2	0.88	0.88	0.88	0.97	0.90	0.97	0.97	0.95	0.96	0.96
6	L1 vs. H1	C2 vs. H2	0.83	0.89	0.84	0.96	0.88	0.98	0.96	0.94	0.96	0.96
7	C1 vs. H1	E2 vs. H2	0.32	0.47	0.55	0.62	0.49	0.77	0.73	0.43	0.46	0.60
8	C1 vs. H1	L2 vs. H2	0.86	0.91	0.85	0.94	0.89	0.97	0.94	0.94	0.94	0.95
9	C1 vs. H1	C2 vs. H2	0.82	0.86	0.79	0.93	0.85	0.93	0.94	0.93	0.94	0.94
10	E2 vs. H2	E2 vs. H2	0.97	0.97	0.94	0.97	0.96	0.99	0.99	0.97	0.99	0.99
11	E2 vs. H2	E1 vs. H1	0.99	1	0.95	1	0.99	1	1	1	1	1.00
12	E2 vs. H2	C1 vs. H1	0.99	1	0.96	1	0.99	1	1	1	1	1.00
13	L2 vs. H2	L2 vs. H2	0.97	0.97	0.87	0.97	0.95	0.98	0.97	0.96	0.99	0.98
14	L2 vs. H2	L1 vs. H1	1	1	0.86	1	0.97	0.99	1	0.89	1	0.97
15	L2 vs. H2	C1 vs. H1	1	1	0.86	1	0.97	0.95	1	0.88	1	0.96
16	C2 vs. H2	E1 vs. H1	0.98	0.99	0.76	1	0.93	0.85	0.91	0.56	0.89	0.80
17	C2 vs. H2	L1 vs. H1	1	1	0.88	1	0.97	0.98	1	0.91	1	0.97
18	C2 vs. H2	C1 vs. H1	1	1	0.88	1	0.97	1	1	0.88	1	0.97

**Table 5:** The comparison of positive predicted value (PPV) between CA-125 alone and all four proteins (CA-125, MIF, OPN, and PROL) when using the four classification techniques, namely K-Nearest Neighbor (KNN), Logistic Regression (LR), Random Forest (RF), and Support Vector Machine (SVM). The PPV measures the liability of a positive test result and is defined as the ratio of the number of condition samples correctly identified to the total number of positive test results. We highlighted the cell(s) with best PPV in each experiment. In general, using logistic regression and all four proteins yield the best results on most occasions.

ID	Training	Testing	CA-125					4 Proteins				
			KNN	LR	RF	SVM	Mean	KNN	LR	RF	SVM	Mean
1	E1 vs. H1	E1 vs. H1	1	1	0.42	1	0.86	1	1	1	1	1.00
2	E1 vs. H1	E2 vs. H2	0.32	0.32	0.24	0.43	0.33	0.87	0.92	0.70	0.61	0.78
3	E1 vs. H1	C2 vs. H2	0.88	0.88	0.76	0.95	0.87	1	0.97	0.94	0.91	0.96
4	L1 vs. H1	L1 vs. H1	1	0.91	1	1	0.98	1	1	1	1	1.00
5	L1 vs. H1	L2 vs. H2	0.63	0.63	0.63	0.86	0.69	0.89	0.92	0.82	0.87	0.88
6	L1 vs. H1	C2 vs. H2	0.62	0.70	0.62	0.85	0.70	0.93	0.90	0.85	0.88	0.89
7	C1 vs. H1	E2 vs. H2	0.10	0.12	0.12	0.15	0.12	0.26	0.24	0.13	0.14	0.19
8	C1 vs. H1	L2 vs. H2	0.61	0.69	0.59	0.76	0.66	0.88	0.79	0.81	0.80	0.82
9	C1 vs. H1	C2 vs. H2	0.63	0.67	0.57	0.76	0.66	0.82	0.84	0.83	0.85	0.84
10	E2 vs. H2	E2 vs. H2	0.33	0.33	0.17	0.33	0.29	0.67	0.80	0.60	0.67	0.69
11	E2 vs. H2	E1 vs. H1	0.83	1	0.60	1	0.86	1	1	1	1	1.00
12	E2 vs. H2	C1 vs. H1	0.97	1	0.91	1	0.97	1	1	1	1	1.00
13	L2 vs. H2	L2 vs. H2	0.87	0.85	0.58	0.85	0.79	0.94	0.89	0.85	0.94	0.91
14	L2 vs. H2	L1 vs. H1	1	1	0.79	1	0.95	0.98	1	0.85	1	0.96
15	L2 vs. H2	C1 vs. H1	1	1	0.83	1	0.96	0.94	1	0.87	1	0.95
16	C2 vs. H2	E1 vs. H1	0.86	0.92	0.41	1	0.80	0.59	0.71	0.33	0.65	0.57
17	C2 vs. H2	L1 vs. H1	1	1	0.81	1	0.95	0.96	1	0.88	1	0.96
18	C2 vs. H2	C1 vs. H1	1	1	0.85	1	0.96	1	1	0.87	1	0.97

**Table 6:** The comparison of negative predicted value (NPV) between CA-125 alone and all four proteins (CA-125, MIF, OPN, and PROL) when using four classification techniques, namely K-Nearest Neighbor (KNN), Logistic Regression (LR), Random Forest (RF), and Support Vector Machine (SVM), in various experiments. The NPV measures the liability of a negative test result and is defined as the ratio of the number of control samples correctly identified to the total number of negative test results. We highlighted the cell(s) with best NPV in each experiment. In general, using random forest and all four proteins yield the best results on most occasions.

ID	Training	Testing	CA-125					4 Proteins				
			KNN	LR	RF	SVM	Mean	KNN	LR	RF	SVM	Mean
1	E1 vs. H1	E1 vs. H1	0.94	0.97	0.93	0.94	0.95	0.97	0.91	1	0.94	0.96
2	E1 vs. H1	E2 vs. H2	0.96	0.96	0.95	0.95	0.96	0.97	0.96	0.97	0.97	0.97
3	E1 vs. H1	C2 vs. H2	0.84	0.85	0.85	0.83	0.84	0.83	0.81	0.87	0.88	0.85
4	L1 vs. H1	L1 vs. H1	0.86	0.94	0.94	0.86	0.90	1	1	1	1	1.00
5	L1 vs. H1	L2 vs. H2	0.89	0.89	0.89	0.89	0.89	0.91	0.92	0.93	0.92	0.92
6	L1 vs. H1	C2 vs. H2	0.86	0.86	0.86	0.85	0.86	0.89	0.91	0.91	0.91	0.91
7	C1 vs. H1	E2 vs. H2	0.94	0.95	0.94	0.95	0.95	0.94	0.95	0.98	0.96	0.96
8	C1 vs. H1	L2 vs. H2	0.91	0.90	0.89	0.89	0.90	0.91	0.92	0.93	0.92	0.92
9	C1 vs. H1	C2 vs. H2	0.89	0.88	0.86	0.86	0.87	0.89	0.91	0.91	0.91	0.91
10	E2 vs. H2	E2 vs. H2	0.93	0.93	0.93	0.93	0.93	0.94	0.96	0.95	0.94	0.95
11	E2 vs. H2	E1 vs. H1	0.86	0.85	0.86	0.85	0.86	0.93	0.96	0.98	0.96	0.96
12	E2 vs. H2	C1 vs. H1	0.64	0.63	0.65	0.63	0.64	0.62	0.70	0.72	0.78	0.71
13	L2 vs. H2	L2 vs. H2	0.88	0.86	0.87	0.94	0.89	0.91	0.91	0.92	0.91	0.91
14	L2 vs. H2	L1 vs. H1	0.87	0.86	0.84	0.86	0.86	0.93	0.92	0.96	0.92	0.93
15	L2 vs. H2	C1 vs. H1	0.80	0.78	0.77	0.78	0.78	0.90	0.85	0.93	0.89	0.89
16	C2 vs. H2	E1 vs. H1	0.93	0.92	0.92	0.91	0.92	0.99	0.99	0.98	0.99	0.99
17	C2 vs. H2	L1 vs. H1	0.86	0.86	0.85	0.85	0.86	0.92	0.92	0.95	0.92	0.93
18	C2 vs. H2	C1 vs. H1	0.78	0.78	0.80	0.77	0.78	0.91	0.92	0.92	0.90	0.91

**Table 7:** The comparison of the area under the ROC (AUC) between CA-125 alone and all four proteins (CA-125, MIF, OPN, and PROL) when using three classification techniques, namely K-Nearest Neighbor (KNN), Logistic Regression (LR), and Random Forest (RF) (the AUC is not available for Support Vector Machine), in various experiments. The AUC measures the performance of the model across all possible classification thresholds. Since SVM has a fixed classification threshold, the AUC is not useful and hence removed from the comparison. We highlighted the cell(s) with best in each experiment. In general, using random forest and all four proteins yield the best results on most occasions.

ID	Training	Testing	CA-125				4 Proteins			
			KNN	LR	RF	Mean	KNN	LR	RF	Mean
1	E1 vs. H1	E1 vs. H1	0.891	0.866	0.866	0.874	1	0.973	1	0.991
2	E1 vs. H1	E2 vs. H2	0.786	0.731	0.781	0.766	0.936	0.942	0.912	0.930
3	E1 vs. H1	C2 vs. H2	0.825	0.807	0.812	0.815	0.847	0.839	0.876	0.854
4	L1 vs. H1	L1 vs. H1	0.961	0.974	0.967	0.967	1	1	1	1.000
5	L1 vs. H1	L2 vs. H2	0.855	0.831	0.863	0.850	0.844	0.850	0.891	0.862
6	L1 vs. H1	C2 vs. H2	0.781	0.807	0.785	0.791	0.874	0.857	0.901	0.877
7	C1 vs. H1	E2 vs. H2	0.714	0.731	0.692	0.712	0.882	0.878	0.848	0.869
8	C1 vs. H1	L2 vs. H2	0.849	0.831	0.840	0.840	0.858	0.846	0.878	0.861
9	C1 vs. H1	C2 vs. H2	0.791	0.807	0.783	0.794	0.830	0.854	0.895	0.860
10	E2 vs. H2	E2 vs. H2	0.481	0.615	0.471	0.522	0.912	0.837	0.882	0.877
11	E2 vs. H2	E1 vs. H1	0.747	0.906	0.749	0.801	0.972	0.991	0.986	0.983
12	E2 vs. H2	C1 vs. H1	0.784	0.953	0.787	0.841	0.904	0.908	0.954	0.922
13	L2 vs. H2	L2 vs. H2	0.838	0.883	0.823	0.848	0.883	0.853	0.934	0.890
14	L2 vs. H2	L1 vs. H1	0.936	0.968	0.919	0.941	0.981	0.996	0.971	0.983
15	L2 vs. H2	C1 vs. H1	0.922	0.953	0.905	0.927	0.959	0.988	0.956	0.968
16	C2 vs. H2	E1 vs. H1	0.926	0.906	0.881	0.904	0.977	0.976	0.949	0.967
17	C2 vs. H2	L1 vs. H1	0.902	0.968	0.905	0.925	0.969	0.998	0.977	0.981
18	C2 vs. H2	C1 vs. H1	0.895	0.953	0.894	0.914	0.967	0.994	0.969	0.977

**Table 8:** Stage and histopathologic diagnosis of patient population.

Histology	Early Stage (I/II)	Late Stage (III/IV)	Number (*n*)
Serous	13	51	64
Endometrioid	2	2	4
Clear Cell	0	1	1
Mucinous	4	2	6
Squamous cell	1	2	3
Granulosa Cell	1	0	1
Other	1	5	6
Unknown	15	53	68
Total	37	116	153

## Data Availability

The data presented in this study are available on request from the corresponding author.

## Supplementary Materials

The following are available online at https://www.mdpi.com/2072-6694/13/2/325/s1, Table S1: Confusion matrices when applying KNN to identify the samples of patients in early stage cancer and healthy patients in data set 1 (E1 vs. H1), using only CA-125 (left panel) and all four proteins (right panel). The samples from patients in early stage cancer and healthy patients in data set 1 (E1 vs. H1) were used to train the model. Note that in this experiment the cohort of early stage cancer samples and healthy samples in data set 1 was divided into 60% for training and 40% for testing. This experiment is corresponding to experiment ID 1 in Table 3, Table S2: Confusion matrices when applying logistic regression to identify the samples of patients in ear-ly stage and healthy patients in data set 1 (E1 vs. H1), using only CA-125 (left panel) and all four proteins (right panel). The samples from patients in early stage cancer and healthy patients in data set 1 (E1 vs. H1) were used to train the model. Note that in this experiment the cohort of early stage cancer samples and healthy samples in data set 1 was divided into 60% for training and 40% for testing. This experiment is corresponding to experiment ID 1 in Table 3, Table S3: Confusion matrices when applying random forest to identify the samples of patients in early stage and healthy patients in data set 1 (E1 vs. H1), using only CA-125 (left panel) and all four proteins (right panel). The samples from patients in early stage cancer and healthy patients in data set 1 (E1 vs. H1) were used to train the model. Note that in this experiment the cohort of early stage cancer samples and healthy samples in data set 1 was divided into 60% for training and 40% for testing. This experiment is corresponding to experiment ID 1 in Table 3, Table S4: Confusion matrices when applying SVM to identify the samples of patients in early stage and healthy patients in data set 1 (E1 vs. H1), using only CA-125 (left panel) and all four proteins (right panel). The samples from patients in early stage cancer and healthy patients in data set 1 (E1 vs. H1) were used to train the model. Note that in this experiment the cohort of early stage cancer samples and healthy samples in data set 1 was divided into 60% for training and 40% for testing. This experiment is corresponding to experiment ID 1 in Table 3, Table S5: Confusion matrices when applying KNN to identify the samples of patients in early stage and healthy pa-tients in data set 2 (E2 vs. H2), using only CA-125 (left panel) and all four proteins (right panel). The samples from patients in early stage cancer and healthy patients in data set 1 (E1 vs. H1) were used to train the model. This experiment is corresponding to experiment ID 2 in Table 3, Table S6: Confusion matrices when applying logistic regression to identify the samples of pa-tients in early stage and healthy patients in data set 2 (E2 vs. H2), using only CA-125 (left panel) and all four proteins (right panel). The samples from patients in early stage cancer and healthy patients in data set 1 (E1 vs. H1) were used to train the model. This experiment is corresponding to experiment ID 2 in Table 3, Table S7: Confusion matrices when applying random forest to identify the samples of patients in early stage and healthy patients in data set 2 (E2 vs. H2), using only CA-125 (left panel) and all four proteins (right panel). The samples from patients in early stage cancer and healthy patients in data set 1 (E1 vs. H1) were used to train the model. This ex-periment is corresponding to experiment ID 2 in Table 3, Table S8: Confusion matrices when ap-plying SVM to identify the samples of patients in early stage and healthy patients in data set 2 (E2 vs. H2), using only CA-125 (left panel) and all four proteins (right panel). The samples from patients in early stage cancer and healthy patients in data set 1 (E1 vs. H1) were used to train the model. This experiment is corresponding to experiment ID 2 in Table 3, Table S9: Confusion ma-trices when applying KNN to identify the samples of ovarian cancer patients and healthy pa-tients in data set 2 (C2 vs. H2), using only CA-125 (left panel) and all four proteins (right panel). The samples from patients in early stage cancer and healthy patients in data set 1 (E1 vs. H1) were used to train the model. This experiment is corresponding to experiment ID 3 in Table 3, Table S10: Confusion matrices when applying logistic regression to identify the samples of ovarian cancer patients and healthy patients in data set 2 (C2 vs. H2), using only CA-125 (left panel) and all four proteins (right panel). The samples from patients in early stage cancer and healthy patients in data set 1 (E1 vs. H1) were used to train the model. This experiment is corre-sponding to experiment ID 3 in Table 3, Table S11: Confusion matrices when applying random forest to identify the samples of ovarian cancer patients and healthy patients in data set 2 (C2 vs. H2), using only CA-125 (left panel) and all four proteins (right panel). The samples from patients in early stage cancer and healthy patients in data set 1 (E1 vs. H1) were used to train the model. This experiment is corresponding to experiment ID 3 in Table 3, Table S12: Confusion matrices when applying SVM to identify the samples of ovarian cancer patients and healthy patients in data set 2 (C2 vs. H2), using only CA-125 (left panel) and all four proteins (right panel). The samples from patients in early stage cancer and healthy patients in data set 1 (E1 vs. H1) were used to train the model. This experiment is corresponding to experiment ID 3 in Table 3, Table S13: Confusion matrices when applying KNN to identify the samples of patients in late stage and healthy patients in data set 1 (L1 vs. H1), using only CA-125 (left panel) and all four proteins (right panel). The samples from patients in late stage cancer and healthy patients in data set 1 (L1 vs. H1) were used to train the model. Note that in this experiment the cohort of late stage cancer samples and healthy samples in data set 1 was divided into 60% for training and 40% for testing. This experiment is corresponding to experiment ID 4 in Table 3, Table S14: Confusion matrices when applying logistic regression to identify the samples of patients in late stage and healthy patients in data set 1 (L1 vs. H1), using only CA-125 (left panel) and all four proteins (right pan-el). The samples from patients in late stage cancer and healthy patients in data set 1 (L1 vs. H1) were used to train the model. Note that in this experiment the cohort of late stage cancer sam-ples and healthy samples in data set 1 was divided into 60% for training and 40% for testing. This experiment is corresponding to experiment ID 4 in Table 3, Table S15: Confusion matrices when applying random forest to identify the samples of patients in late stage and healthy patients in data set 1 (L1 vs. H1), using only CA-125 (left panel) and all four proteins (right panel). The sam-ples from patients in late stage cancer and healthy patients in data set 1 (L1 vs. H1) were used to train the model. Note that in this experiment the cohort of late stage cancer samples and healthy samples in data set 1 was divided into 60% for training and 40% for testing. This experiment is corresponding to experiment ID 4 in Table 3, Table S16: Confusion matrices when applying SVM to identify the samples of patients in late stage and healthy patients in data set 1 (L1 vs. H1), us-ing only CA-125 (left panel) and all four proteins (right panel). The samples from patients in late stage cancer and healthy patients in data set 1 (L1 vs. H1) were used to train the model. Note that in this experiment the cohort of late stage cancer samples and healthy samples in data set 1 was divided into 60% for training and 40% for testing. This experiment is corresponding to experi-ment ID 4 in Table 3, Table S17: Confusion matrices when applying KNN to identify the samples of patients in late stage and healthy patients in data set 2 (L2 vs. H2), using only CA-125 (left panel) and all four proteins (right panel). The samples from patients in late stage cancer and healthy patients in data set 1 (L1 vs. H1) were used to train the model. This experiment is corre-sponding to experiment ID 5 in Table 3, Table S18: Confusion matrices when applying logistic regression to identify the samples of patients in late stage and healthy patients in data set 2 (L2 vs. H2), using only CA-125 (left panel) and all four proteins (right panel). The samples from pa-tients in late stage cancer and healthy patients in data set 1 (L1 vs. H1) were used to train the model. This experiment is corresponding to experiment ID 5 in Table 3, Table S19: Confusion matrices when applying random forest to identify the samples of patients in late stage and healthy patients in data set 2 (L2 vs. H2), using only CA-125 (left panel) and all four proteins (right panel). The samples from patients in late stage cancer and healthy patients in data set 1 (L1 vs. H1) were used to train the model. This experiment is corresponding to experiment ID 5 in Table 3, Table S20: Confusion matrices when applying SVM to identify the samples of patients in late stage and healthy patients in data set 2 (L2 vs. H2), using only CA-125 (left panel) and all four proteins (right panel). The samples from patients in late stage cancer and healthy patients in data set 1 (L1 vs. H1) were used to train the model. This experiment is corresponding to experi-ment ID 5 in Table 3, Table S21: Confusion matrices when applying KNN to identify the samples of ovarian cancer patients and healthy patients in data set 2 (C2 vs. H2), using only CA-125 (left panel) and all four proteins (right panel). The samples from patients in late stage cancer and healthy patients in data set 1 (L1 vs. H1) were used to train the model. This experiment is corre-sponding to experiment ID 6 in Table 3, Table S22: Confusion matrices when applying logistic regression to identify the samples of ovarian cancer patients and healthy patients in data set 2 (C2 vs. H2), using only CA-125 (left panel) and all four proteins (right panel). The samples from patients in late stage cancer and healthy patients in data set 1 (L1 vs. H1) were used to train the model. This experiment is corresponding to experiment ID 6 in Table 3, Table S23: Confusion matrices when applying random forest to identify the samples of ovarian cancer patients and healthy patients in data set 2 (C2 vs. H2), using only CA-125 (left panel) and all four proteins (right panel). The samples from patients in late stage cancer and healthy patients in data set 1 (L1 vs. H1) were used to train the model. This experiment is corresponding to experiment ID 6 in Table 3, Table S24: Confusion matrices when applying SVM to identify the samples of ovarian cancer patients and healthy patients in data set 2 (C2 vs. H2), using only CA-125 (left panel) and all four proteins (right panel). The samples from patients in late stage cancer and healthy pa-tients in data set 1 (L1 vs. H1) were used to train the model. This experiment is corresponding to experiment ID 6 in Table 3, Table S25: Confusion matrices when applying KNN to identify the samples of patients in early stage and healthy patients in data set 2 (E2 vs. H2), using only CA-125 (left panel) and all four proteins (right panel). The samples from ovarian cancer patients and healthy patients in data set 1 (C1 vs. H1) were used to train the model. This experiment is corresponding to experiment ID 7 in Table 3, Table S26: Confusion matrices when applying lo-gistic regression to identify the samples of patients in early stage and healthy patients in data set 2 (E2 vs. H2), using only CA-125 (left panel) and all four proteins (right panel). The samples from ovarian cancer patients and healthy patients in data set 1 (C1 vs. H1) were used to train the mod-el. This experiment is corresponding to experiment ID 7 in Table 3, Table S27: Confusion matri-ces when applying random forest to identify the samples of patients in early stage and healthy patients in data set 2 (E2 vs. H2), using only CA-125 (left panel) and all four proteins (right pan-el). The samples from ovarian cancer patients and healthy patients in data set 1 (C1 vs. H1) were used to train the model. This experiment is corresponding to experiment ID 7 in Table 3, Table S28: Confusion matrices when applying SVM to identify the samples of patients in early stage and healthy patients in data set 2 (E2 vs. H2), using only CA-125 (left panel) and all four proteins (right panel). The samples from ovarian cancer patients and healthy patients in data set 1 (C1 vs. H1) were used to train the model. This experiment is corresponding to experiment ID 7 in Table 3, Table S29: Confusion matrices when applying KNN to identify the samples of patients in late stage and healthy patients in data set 2 (L2 vs. H2), using only CA-125 (left panel) and all four proteins (right panel). The samples from ovarian cancer patients and healthy patients in data set 1 (C1 vs. H1) were used to train the model. This experiment is corresponding to experiment ID 8 in Table 3, Table S30: Confusion matrices when applying logistic regression to identify the sam-ples of patients in late stage and healthy patients in data set 2 (L2 vs. H2), using only CA-125 (left panel) and all four proteins (right panel). The samples from ovarian cancer patients and healthy patients in data set 1 (C1 vs. H1) were used to train the model. This experiment is corresponding to experiment ID 8 in Table 3, Table S31: Confusion matrices when applying random forest to identify the samples of patients in late stage and healthy patients in data set 2 (L2 vs. H2), using only CA-125 (left panel) and all four proteins (right panel). The samples from ovarian cancer pa-tients and healthy patients in data set 1 (C1 vs. H1) were used to train the model. This experi-ment is corresponding to experiment ID 8 in Table 3, Table S32: Confusion matrices when ap-plying SVM to identify the samples of patients in late stage and healthy patients in data set 2 (L2 vs. H2), using only CA-125 (left panel) and all four proteins (right panel). The samples from ovarian cancer patients and healthy patients in data set 1 (C1 vs. H1) were used to train the mod-el. This experiment is corresponding to experiment ID 8 in Table 3, Table S33: Confusion matri-ces when applying KNN to identify the samples of ovarian cancer patients and healthy patients in data set 2 (C2 vs. H2), using only CA-125 (left panel) and all four proteins (right panel). The samples from ovarian cancer patients and healthy patients in data set 1 (C1 vs. H1) were used to train the model. This experiment is corresponding to experiment ID 9 in Table 3, Table S34: Confusion matrices when applying logistic regression to identify the samples of ovarian cancer patients and healthy patients in data set 2 (C2 vs. H2), using only CA-125 (left panel) and all four proteins (right panel). The samples from ovarian cancer patients and healthy patients in data set 1 (C1 vs. H1) were used to train the model. This experiment is corresponding to experiment ID 9 in Table 3, Table S35: Confusion matrices when applying random forest to identify the samples of ovarian cancer patients and healthy patients in data set 2 (C2 vs. H2), using only CA-125 (left panel) and all four proteins (right panel). The samples from ovarian cancer patients and healthy patients in data set 1 (C1 vs. H1) were used to train the model. This experiment is corresponding to experiment ID 9 in Table 3, Table S36: Confusion matrices when applying SVM to identify the samples of ovarian cancer patients and healthy patients in data set 2 (C2 vs. H2), using only CA-125 (left panel) and all four proteins (right panel). The samples from ovarian cancer patients and healthy patients in data set 1 (C1 vs. H1) were used to train the model. This experiment is corresponding to experiment ID 9 in Table 3, Table S37: Confusion matrices when applying KNN to identify the samples of patients in early stage and healthy patients in data set 2 (E2 vs. H2), using only CA-125 (left panel) and all four proteins (right panel). The samples from patients in early stage cancer and healthy patients in data set 2 (E2 vs. E2) were used to train the model. Note that in this experiment the cohort of early stage cancer samples and healthy samples in da-ta set 2 was divided into 60% for training and 40% for testing. This experiment is corresponding to experiment ID 9 in Table 3. This experiment is corresponding to experiment ID 10 in Table 3, Table S38: Confusion matrices when applying logistic regression to identify the samples of pa-tients in early stage and healthy patients in data set 2 (E2 vs. H2), using only CA-125 (left panel) and all four proteins (right panel). The samples from patients in early stage cancer and healthy patients in data set 2 (E2 vs. E2) were used to train the model. Note that in this experiment the cohort of early stage cancer samples and healthy samples in data set 2 was divided into 60% for training and 40% for testing. This experiment is corresponding to experiment ID 10 in Table 3, Table S39: Confusion matrices when applying random forest to identify the samples of patients in early stage and healthy patients in data set 2 (E2 vs. H2), using only CA-125 (left panel) and all four proteins (right panel). The samples from patients in early stage cancer and healthy patients in data set 2 (E2 vs. E2) were used to train the model. Note that in this experiment the cohort of early stage cancer samples and healthy samples in data set 2 was divided into 60% for training and 40% for testing. This experiment is corresponding to experiment ID 10 in Table 3, Table S40: Confusion matrices when applying SVM to identify the samples of patients in early stage and healthy patients in data set 2 (E2 vs. H2), using only CA-125 (left panel) and all four proteins (right panel). The samples from patients in early stage cancer and healthy patients in data set 2 (E2 vs. E2) were used to train the model. Note that in this experiment the cohort of early stage cancer samples and healthy samples in data set 2 was divided into 60% for training and 40% for testing. This experiment is corresponding to experiment ID 10 in Table 3, Table S41: Confusion matrices when applying KNN to identify the samples of patients in early stage and healthy pa-tients in data set 1 (E1 vs. H1), using only CA-125 (left panel) and all four proteins (right panel). The samples from patients in early stage cancer and healthy patients in data set 2 (E2 vs. E2) were used to train the model. This experiment is corresponding to experiment ID 11 in Table 3, Table S42: Confusion matrices when applying logistic regression to identify the samples of patients in early stage and healthy patients in data set 1 (E1 vs. H1), using only CA-125 (left panel) and all four proteins (right panel). The samples from patients in early stage cancer and healthy patients in data set 2 (E2 vs. E2) were used to train the model. This experiment is corresponding to ex-periment ID 11 in Table 3, Table S43: Confusion matrices when applying random forest to iden-tify the samples of patients in early stage and healthy patients in data set 1 (E1 vs. H1), using only CA-125 (left panel) and all four proteins (right panel). The samples from patients in early stage cancer and healthy patients in data set 2 (E2 vs. E2) were used to train the model. This ex-periment is corresponding to experiment ID 11 in Table 3, Table S44: Confusion matrices when applying SVM to identify the samples of patients in early stage and healthy patients in data set 1 (E1 vs. H1), using only CA-125 (left panel) and all four proteins (right panel). The samples from patients in early stage cancer and healthy patients in data set 2 (E2 vs. E2) were used to train the model. This experiment is corresponding to experiment ID 11 in Table 3, Table S45: Confusion matrices when applying KNN to identify the samples of ovarian cancer patients and healthy pa-tients in data set 1 (C1 vs. H1), using only CA-125 (left panel) and all four proteins (right panel). The samples from patients in early stage cancer and healthy patients in data set 2 (E2 vs. E2) were used to train the model. This experiment is corresponding to experiment ID 12 in Table 3, Table S46: Confusion matrices when applying logistic regression to identify the samples of ovarian cancer patients and healthy patients in data set 1 (C1 vs. H1), using only CA-125 (left panel) and all four proteins (right panel). The samples from patients in early stage cancer and healthy pa-tients in data set 2 (E2 vs. E2) were used to train the model. This experiment is corresponding to experiment ID 12 in Table 3, Table S47: Confusion matrices when applying random forest to identify the samples of ovarian cancer patients and healthy patients in data set 1 (C1 vs. H1), us-ing only CA-125 (left panel) and all four proteins (right panel). The samples from patients in early stage cancer and healthy patients in data set 2 (E2 vs. E2) were used to train the model. This experiment is corresponding to experiment ID 12 in Table 3, Table S48: Confusion matrices when applying SVM to identify the samples of ovarian cancer patients and healthy patients in data set 1 (C1 vs. H1), using only CA-125 (left panel) and all four proteins (right panel). The samples from patients in early stage cancer and healthy patients in data set 2 (E2 vs. E2) were used to train the model. This experiment is corresponding to experiment ID 12 in Table 3, Table S49: Confusion matrices when applying KNN to identify the samples of patients in late stage and healthy patients in data set 2 (L2 vs. H2), using only CA-125 (left panel) and all four proteins (right panel). The samples from patients in late stage cancer and healthy patients in data set 2 (L2 vs. L2) were used to train the model. Note that in this experiment the cohort of late stage cancer samples and healthy samples in data set 2 was divided into 60% for training and 40% for testing. This experiment is corresponding to experiment ID 13 in Table 3. Table S50: Confusion matrices when applying logistic regression to identify the samples of patients in late stage and healthy patients in data set 2 (L2 vs. H2), using only CA-125 (left panel) and all four proteins (right pan-el). The samples from patients in late stage cancer and healthy patients in data set 2 (L2 vs. L2) were used to train the model. Note that in this experiment the cohort of late stage cancer sam-ples and healthy samples in data set 2 was divided into 60% for training and 40% for testing. This experiment is corresponding to experiment ID 13 in Table 3. Table S51: Confusion matrices when applying random forest to identify the samples of patients in late stage and healthy patients in data set 2 (L2 vs. H2), using only CA-125 (left panel) and all four proteins (right panel). The sam-ples from patients in late stage cancer and healthy patients in data set 2 (L2 vs. L2) were used to train the model. Note that in this experiment the cohort of late stage cancer samples and healthy samples in data set 2 was divided into 60% for training and 40% for testing. This experiment is corresponding to experiment ID 13 in Table 3, Table S52: Confusion matrices when applying SVM to identify the samples of patients in late stage and healthy patients in data set 2 (L2 vs. H2), using only CA-125 (left panel) and all four proteins (right panel). The samples from patients in late stage cancer and healthy patients in data set 2 (L2 vs. L2) were used to train the model. Note that in this experiment the cohort of late stage cancer samples and healthy samples in data set 2 was divided into 60% for training and 40% for testing. This experiment is corresponding to experiment ID 13 in Table 3, Table S53: Confusion matrices when applying KNN to identify the samples of patients in late stage and healthy patients in data set 1 (L1 vs. H1), using only CA-125 (left panel) and all four proteins (right panel). The samples from patients in late stage cancer and healthy patients in data set 2 (L2 vs. L2) were used to train the model. This experiment is corre-sponding to experiment ID 14 in Table 3, Table S54: Confusion matrices when applying logistic regression to identify the samples of patients in late stage and healthy patients in data set 1 (L1 vs. H1), using only CA-125 (left panel) and all four proteins (right panel). The samples from pa-tients in late stage cancer and healthy patients in data set 2 (L2 vs. L2) were used to train the model. This experiment is corresponding to experiment ID 14 in Table 3, Table S55: Confusion matrices when applying random forest to identify the samples of patients in late stage and healthy patients in data set 1 (L1 vs. H1), using only CA-125 (left panel) and all four proteins (right panel). The samples from patients in late stage cancer and healthy patients in data set 2 (L2 vs. L2) were used to train the model. This experiment is corresponding to experiment ID 14 in Table 3, Table S56: Confusion matrices when applying SVM to identify the samples of patients in late stage and healthy patients in data set 1 (L1 vs. H1), using only CA-125 (left panel) and all four proteins (right panel). The samples from patients in late stage cancer and healthy patients in data set 2 (L2 vs. L2) were used to train the model. This experiment is corresponding to experi-ment ID 14 in Table 3, Table S57: Confusion matrices when applying KNN to identify the sam-ples of ovarian cancer patients and healthy patients in data set 1 (C1 vs. H1), using only CA-125 (left panel) and all four proteins (right panel). The samples from patients in late stage cancer and healthy patients in data set 2 (L2 vs. L2) were used to train the model. This experiment is corre-sponding to experiment ID 15 in Table 3, Table S58: Confusion matrices when applying logistic regression to identify the samples of ovarian cancer patients and healthy patients in data set 1 (C1 vs. H1), using only CA-125 (left panel) and all four proteins (right panel). The samples from patients in late stage cancer and healthy patients in data set 2 (L2 vs. L2) were used to train the model. This experiment is corresponding to experiment ID 15 in Table 3, Table S59: Confusion matrices when applying random forest to identify the samples of ovarian cancer patients and healthy patients in data set 1 (C1 vs. H1), using only CA-125 (left panel) and all four proteins (right panel). The samples from patients in late stage cancer and healthy patients in data set 2 (L2 vs. L2) were used to train the model. This experiment is corresponding to experiment ID 15 in Table 3, Table S60: Confusion matrices when applying SVM to identify the samples of ovarian cancer patients and healthy patients in data set 1 (C1 vs. H1), using only CA-125 (left panel) and all four proteins (right panel). The samples from patients in late stage cancer and healthy pa-tients in data set 2 (L2 vs. L2) were used to train the model. This experiment is corresponding to experiment ID 15 in Table 3, Table S61: Confusion matrices when applying KNN to identify the samples of patients in early stage and healthy patients in data set 1 (E1 vs. H1), using only CA-125 (left panel) and all four proteins (right panel). The samples from ovarian cancer patients and healthy patients in data set 2 (C2 vs. H2) were used to train the model. This experiment is corresponding to experiment ID 16 in Table 3, Table S62: Confusion matrices when applying lo-gistic regression to identify the samples of patients in early stage and healthy patients in data set 1 (E1 vs. H1), using only CA-125 (left panel) and all four proteins (right panel). The samples from ovarian cancer patients and healthy patients in data set 2 (C2 vs. H2) were used to train the mod-el. This experiment is corresponding to experiment ID 16 in Table 3, Table S63: Confusion ma-trices when applying random forest to identify the samples of patients in early stage and healthy patients in data set 1 (E1 vs. H1), using only CA-125 (left panel) and all four proteins (right pan-el). The samples from ovarian cancer patients and healthy patients in data set 2 (C2 vs. H2) were used to train the model. This experiment is corresponding to experiment ID 16 in Table 3, Table S64: Confusion matrices when applying SVM to identify the samples of patients in early stage and healthy patients in data set 1 (E1 vs. H1), using only CA-125 (left panel) and all four proteins (right panel). The samples from ovarian cancer patients and healthy patients in data set 2 (C2 vs. H2) were used to train the model. This experiment is corresponding to experiment ID 16 in Table 3, Table S65: Confusion matrices when applying KNN to identify the samples of patients in late stage and healthy patients in data set 1 (L1 vs. H1), using only CA-125 (left panel) and all four proteins (right panel). The samples from ovarian cancer patients and healthy patients in data set 2 (C2 vs. H2) were used to train the model. This experiment is corresponding to experiment ID 17 in Table 3, Table S66: Confusion matrices when applying logistic regression to identify the samples of patients in late stage and healthy patients in data set 1 (L1 vs. H1), using only CA-125 (left panel) and all four proteins (right panel). The samples from ovarian cancer patients and healthy patients in data set 2 (C2 vs. H2) were used to train the model. This experiment is corre-sponding to experiment ID 17 in Table 3, Table S67: Confusion matrices when applying random forest to identify the samples of patients in late stage and healthy patients in data set 1 (L1 vs. H1), using only CA-125 (left panel) and all four proteins (right panel). The samples from ovarian cancer patients and healthy patients in data set 2 (C2 vs. H2) were used to train the model. This experiment is corresponding to experiment ID 17 in Table 3, Table S68: Confusion matrices when applying SVM to identify the samples of patients in late stage and healthy patients in data set 1 (L1 vs. H1), using only CA-125 (left panel) and all four proteins (right panel). The samples from ovarian cancer patients and healthy patients in data set 2 (C2 vs. H2) were used to train the mod-el. This experiment is corresponding to experiment ID 17 in Table 3, Table S69: Confusion ma-trices when applying KNN to identify the samples of ovarian cancer patients and healthy pa-tients in data set 1 (C1 vs. H1), using only CA-125 (left panel) and all four proteins (right panel). The samples from ovarian cancer patients and healthy patients in data set 2 (C2 vs. H2) were used to train the model. This experiment is corresponding to experiment ID 18 in Table 3, Table S70: Confusion matrices when applying logistic regression to identify the samples of ovarian cancer patients and healthy patients in data set 1 (C1 vs. H1), using only CA-125 (left panel) and all four proteins (right panel). The samples from ovarian cancer patients and healthy patients in data set 2 (C2 vs. H2) were used to train the model. This experiment is corresponding to experi-ment ID 18 in Table 3, Table S71: Confusion matrices when applying random forest to identify the samples of ovarian cancer patients and healthy patients in data set 1 (C1 vs. H1), using only CA-125 (left panel) and all four proteins (right panel). The samples from ovarian cancer patients and healthy patients in data set 2 (C2 vs. H2) were used to train the model. This experiment is corresponding to experiment ID 18 in Table 3, Table S72: Confusion matrices when applying SVM to identify the samples of ovarian cancer patients and healthy patients in data set 1 (C1 vs. H1), using only CA-125 (left panel) and all four proteins (right panel). The samples from ovarian cancer patients and healthy patients in data set 2 (C2 vs. H2) were used to train the model. This experiment is corresponding to experiment ID 18 in Table 3, Figure S1: The AUC and Youden index when applying KNN, logistic regression, and random forest to identify control and healthy samples in experiment 1 in Table 8, using only CA-125 and all four proteins. Figure S2: The AUC and Youden index when applying KNN, logistic regression, and random forest to identify control and healthy samples in experiment 2 in Table 8, using only CA-125 and all four proteins. Figure S3: The AUC and Youden index when applying KNN, logistic regression, and random forest to identify control and healthy samples in experiment 3 in Table 8, using only CA-125 and all four proteins. Figure S4: The AUC and Youden index when applying KNN, lo-gistic regression, and random forest to identify control and healthy samples in experiment 4 in Table 8, using only CA-125 and all four proteins. Figure S5: The AUC and Youden index when applying KNN, logistic regression, and random forest to identify control and healthy samples in experiment 5 in Table 8, using only CA-125 and all four proteins, Figure S6: The AUC and Youden index when applying KNN, logistic regression, and random forest to identify control and healthy samples in experiment 6 in Table 8, using only CA-125 and all four proteins, Figure S7: The AUC and Youden index when applying KNN, logistic regression, and random forest to identify control and healthy samples in experiment 7 in Table 8, using only CA-125 and all four proteins, Figure S8: The AUC and Youden index when applying KNN, logistic regression, and random forest to identify control and healthy samples in experiment 8 in Table 8, using only CA-125 and all four proteins, Figure S9: The AUC and Youden index when applying KNN, lo-gistic regression, and random forest to identify control and healthy samples in experiment 9 in Table 8, using only CA-125 and all four proteins, Figure S10: The AUC and Youden index when applying KNN, logistic regression, and random forest to identify control and healthy samples in experiment 10 in Table 8, using only CA-125 and all four proteins, Figure S11: The AUC and Youden index when applying KNN, logistic regression, and random forest to identify control and healthy samples in experiment 11 in Table 8, using only CA-125 and all four proteins, Figure S12: The AUC and Youden index when applying KNN, logistic regression, and random forest to identify control and healthy samples in experiment 12 in Table 8, using only CA-125 and all four proteins, Figure S13: The AUC and Youden index when applying KNN, logistic regression, and random forest to identify control and healthy samples in experiment 13 in Table 8, using only CA-125 and all four proteins, Figure S14: The AUC and Youden index when applying KNN, lo-gistic regression, and random forest to identify control and healthy samples in experiment 14 in Table 8, using only CA-125 and all four proteins, Figure S15: The AUC and Youden index when applying KNN, logistic regression, and random forest to identify control and healthy samples in experiment 15 in Table 8, using only CA-125 and all four proteins, Figure S16: The AUC and Youden index when applying KNN, logistic regression, and random forest to identify control and healthy samples in experiment 16 in Table 8, using only CA-125 and all four proteins, Figure S17: The AUC and Youden index when applying KNN, logistic regression, and random forest to identify control and healthy samples in experiment 17 in Table 8, using only CA-125 and all four proteins, Figure S18: The AUC and Youden index when applying KNN, logistic regression, and random forest to identify control and healthy samples in experiment 17 in Table 8, using only CA-125 and all four proteins.
