# Supplementary material for: Automated Assay of a Four-Protein Biomarker Panel for Improved Detection of Ovarian Cancer
Source: Cancers (Basel). 2021 Jan 17;13(2):325. doi: 10.3390/cancers13020325 (PMC7830619; doi:10.3390/cancers13020325)
Supplement: Supplementary file 1 [file cancers-13-00325-s001.pdf]

Automated assay of four-protein biomarker panel for improved early detection of ovarian cancer (Supplementary Materials)

January 9, 2021

Table S1: Confusion matrices when applying KNN to identify the samples of patients in early stage cancer and healthy patients in data set 1 (E1 vs H1), using only CA-125 (left panel) and all four proteins (right panel). The samples from patients in early stage cancer and healthy patients in data set 1 (E1 vs H1) were used to train the model. Note that in this experiment the cohort of early stage cancer samples and healthy samples in data set 1 was divided into 60% for training and 40% for testing. This experiment is corresponding to experiment ID 1 in Table 3.

Actual label

		Disease	Healthy	Prevalence 0.18
Predicted	Disease	5	0	PPV 1
	Healthy	2	32	NPV 0.94
		Sensitivity 0.71	Specificity 1	Accuracy 0.95

Using CA-125 alone

Actual label

		Disease	Healthy	Prevalence 0.18
Predicted	Disease	6	0	PPV 1
	Healthy	1	32	NPV 0.97
		Sensitivity 0.86	Specificity 1	Accuracy 0.97

Using four proteins

Table S2: Confusion matrices when applying logistic regression to identify the samples of patients in early stage and healthy patients in data set 1 (E1 vs H1), using only CA-125 (left panel) and all four proteins (right panel). The samples from patients in early stage cancer and healthy patients in data set 1 (E1 vs H1) were used to train the model. Note that in this experiment the cohort of early stage cancer samples and healthy samples in data set 1 was divided into 60% for training and 40% for testing. This experiment is corresponding to experiment ID 1 in Table 3.

Actual label

		Disease	Healthy	Prevalence 0.18
Predicted	Disease	6	0	PPV 1
	Healthy	1	32	NPV 0.97
		Sensitivity 0.86	Specificity 1	Accuracy 0.97

Using CA-125 alone

Actual label

		Disease	Healthy	Prevalence 0.18
Predicted	Disease	4	0	PPV 1
	Healthy	3	32	NPV 0.91
		Sensitivity 0.57	Specificity 1	Accuracy 0.92

Using four proteins

Table S3: Confusion matrices when applying random forest to identify the samples of patients in early stage and healthy patients in data set 1 (E1 vs H1), using only CA-125 (left panel) and all four proteins (right panel). The samples from patients in early stage cancer and healthy patients in data set 1 (E1 vs H1) were used to train the model. Note that in this experiment the cohort of early stage cancer samples and healthy samples in data set 1 was divided into 60% for training and 40% for testing. This experiment is corresponding to experiment ID 1 in Table 3.

Actual label

		Disease	Healthy	Prevalence 0.18
Predicted	Disease	5	7	PPV 0.42
	Healthy	2	25	NPV 0.93
		Sensitivity 0.71	Specificity 0.78	Accuracy 0.77

Using CA-125 alone

Actual label

		Disease	Healthy	Prevalence 0.18
Predicted	Disease	7	0	PPV 1
	Healthy	0	32	NPV 1
		Sensitivity 1	Specificity 1	Accuracy 1

Using four proteins

Table S4: Confusion matrices when applying SVM to identify the samples of patients in early stage and healthy patients in data set 1 (E1 vs H1), using only CA-125 (left panel) and all four proteins (right panel). The samples from patients in early stage cancer and healthy patients in data set 1 (E1 vs H1) were used to train the model. Note that in this experiment the cohort of early stage cancer samples and healthy samples in data set 1 was divided into 60% for training and 40% for testing. This experiment is corresponding to experiment ID 1 in Table 3.

		Actual label		
		Disease	Healthy	Prevalence 0.18
Predicted	Disease	5	0	PPV 1
	Healthy	2	32	NPV 0.94
		Sensitivity 0.71	Specificity 1	Accuracy 0.95

Using CA-125 alone

		Actual label		
		Disease	Healthy	Prevalence 0.18
Predicted	Disease	5	0	PPV 1
	Healthy	2	32	NPV 0.94
		Sensitivity 0.71	Specificity 1	Accuracy 0.95

Using four proteins

Table S5: Confusion matrices when applying KNN to identify the samples of patients in early stage and healthy patients in data set 2 (E2 vs H2), using only CA-125 (left panel) and all four proteins (right panel). The samples from patients in early stage cancer and healthy patients in data set 1 (E1 vs H1) were used to train the model. This experiment is corresponding to experiment ID 2 in Table 3.

		Actual label		
		Disease	Healthy	Prevalence 0.09
Predicted	Disease	11	23	PPV 0.32
	Healthy	8	176	NPV 0.96
		Sensitivity 0.58	Specificity 0.88	Accuracy 0.86

Using CA-125 alone

		Actual label		
		Disease	Healthy	Prevalence 0.09
Predicted	Disease	13	2	PPV 0.87
	Healthy	6	196	NPV 0.97
		Sensitivity 0.68	Specificity 0.99	Accuracy 0.97

Using four proteins

Table S6: Confusion matrices when applying logistic regression to identify the samples of patients in early stage and healthy patients in data set 2 (E2 vs H2), using only CA-125 (left panel) and all four proteins (right panel). The samples from patients in early stage cancer and healthy patients in data set 1 (E1 vs H1) were used to train the model. This experiment is corresponding to experiment ID 2 in Table 3.

		Actual label		
		Disease	Healthy	Prevalence 0.09
Predicted	Disease	12	26	PPV 0.32
	Healthy	7	173	NPV 0.96
		Sensitivity 0.63	Specificity 0.87	Accuracy 0.85

Using CA-125 alone

		Actual label		
		Disease	Healthy	Prevalence 0.09
Predicted	Disease	11	1	PPV 0.92
	Healthy	8	198	NPV 0.96
		Sensitivity 0.58	Specificity 0.99	Accuracy 0.96

Using four proteins

Table S7: Confusion matrices when applying random forest to identify the samples of patients in early stage and healthy patients in data set 2 (E2 vs H2), using only CA-125 (left panel) and all four proteins (right panel). The samples from patients in early stage cancer and healthy patients in data set 1 (E1 vs H1) were used to train the model. This experiment is corresponding to experiment ID 2 in Table 3.

Using CA-125 alone

		Actual label: Disease	Actual label: Healthy	
		Disease	Healthy	Prevalence 0.09
Predicted	Disease	11	35	PPV 0.24
Predicted	Healthy	8	164	NPV 0.95
		Sensitivity 0.58	Specificity 0.82	Accuracy 0.80

Using four proteins

		Actual label: Disease	Actual label: Healthy	
		Disease	Healthy	Prevalence 0.09
Predicted	Disease	14	6	PPV 0.7
Predicted	Healthy	5	193	NPV 0.97
		Sensitivity 0.74	Specificity 0.97	Accuracy 0.95

Table S8: Confusion matrices when applying SVM to identify the samples of patients in early stage and healthy patients in data set 2 (E2 vs H2), using only CA-125 (left panel) and all four proteins (right panel). The samples from patients in early stage cancer and healthy patients in data set 1 (E1 vs H1) were used to train the model. This experiment is corresponding to experiment ID 2 in Table 3.

Using CA-125 alone

		Actual label: Disease	Actual label: Healthy	
		Disease	Healthy	Prevalence 0.09
Predicted	Disease	9	12	PPV 0.43
Predicted	Healthy	10	187	NPV 0.95
		Sensitivity 0.47	Specificity 0.94	Accuracy 0.90

Using four proteins

		Actual label: Disease	Actual label: Healthy	
		Disease	Healthy	Prevalence 0.09
Predicted	Disease	14	9	PPV 0.61
Predicted	Healthy	5	190	NPV 0.97
		Sensitivity 0.74	Specificity 0.95	Accuracy 0.94

Table S9: Confusion matrices when applying KNN to identify the samples of ovarian cancer patients and healthy patients in data set 2 (C2 vs H2), using only CA-125 (left panel) and all four proteins (right panel). The samples from patients in early stage cancer and healthy patients in data set 1 (E1 vs H1) were used to train the model. This experiment is corresponding to experiment ID 3 in Table 3.

Using CA-125 alone

		Actual label: Disease	Actual label: Healthy	
		Disease	Healthy	Prevalence 0.29
Predicted	Disease	44	6	PPV 0.88
Predicted	Healthy	36	193	NPV 0.84
		Sensitivity 0.55	Specificity 0.97	Accuracy 0.85

Using four proteins

		Actual label: Disease	Actual label: Healthy	
		Disease	Healthy	Prevalence 0.29
Predicted	Disease	40	0	PPV 1
Predicted	Healthy	40	199	NPV 0.83
		Sensitivity 0.5	Specificity 1	Accuracy 0.86

Table S10: Confusion matrices when applying logistic regression to identify the samples of ovarian cancer patients and healthy patients in data set 2 (C2 vs H2), using only CA-125 (left panel) and all four proteins (right panel). The samples from patients in early stage cancer and healthy patients in data set 1 (E1 vs H1) were used to train the model. This experiment is corresponding to experiment ID 3 in Table 3.

Using CA-125 alone

Predicted \ Actual label	Disease	Healthy	Prevalence 0.29
Disease	45	6	PPV 0.88
Healthy	35	193	NPV 0.85
	Sensitivity 0.58	Specificity 0.97	Accuracy 0.85

Using four proteins

Predicted \ Actual label	Disease	Healthy	Prevalence 0.29
Disease	34	1	PPV 0.97
Healthy	46	198	NPV 0.81
	Sensitivity 0.42	Specificity 1	Accuracy 0.83

Table S11: Confusion matrices when applying random forest to identify the samples of ovarian cancer patients and healthy patients in data set 2 (C2 vs H2), using only CA-125 (left panel) and all four proteins (right panel). The samples from patients in early stage cancer and healthy patients in data set 1 (E1 vs H1) were used to train the model. This experiment is corresponding to experiment ID 3 in Table 3.

Using CA-125 alone

Predicted \ Actual label	Disease	Healthy	Prevalence 0.29
Disease	47	15	PPV 0.76
Healthy	33	184	NPV 0.85
	Sensitivity 0.59	Specificity 0.92	Accuracy 0.83

Using four proteins

Predicted \ Actual label	Disease	Healthy	Prevalence 0.29
Disease	50	3	PPV 0.94
Healthy	30	196	NPV 0.87
	Sensitivity 0.63	Specificity 0.98	Accuracy 0.88

Table S12: Confusion matrices when applying SVM to identify the samples of ovarian cancer patients and healthy patients in data set 2 (C2 vs H2), using only CA-125 (left panel) and all four proteins (right panel). The samples from patients in early stage cancer and healthy patients in data set 1 (E1 vs H1) were used to train the model. This experiment is corresponding to experiment ID 3 in Table 3.

Using CA-125 alone

Predicted \ Actual label	Disease	Healthy	Prevalence 0.29
Disease	39	2	PPV 0.95
Healthy	41	197	NPV 0.83
	Sensitivity 0.49	Specificity 0.99	Accuracy 0.85

Using four proteins

Predicted \ Actual label	Disease	Healthy	Prevalence 0.29
Disease	53	5	PPV 0.91
Healthy	27	194	NPV 0.88
	Sensitivity 0.66	Specificity 0.97	Accuracy 0.89

Table S13: Confusion matrices when applying KNN to identify the samples of patients in late stage and healthy patients in data set 1 (L1 vs H1), using only CA-125 (left panel) and all four proteins (right panel). The samples from patients in late stage cancer and healthy patients in data set 1 (L1 vs H1) were used to train the model. Note that in this experiment the cohort of late stage cancer samples and healthy samples in data set 1 was divided into 60% for training and 40% for testing. This experiment is corresponding to experiment ID 4 in Table 3.

		Actual label: Disease	Actual label: Healthy	Prevalence 0.41
Predicted	Disease	17	0	PPV 1
Predicted	Healthy	5	32	NPV 0.86
		Sensitivity 0.77	Specificity 1	Accuracy 0.91

Using CA-125 alone

		Actual label: Disease	Actual label: Healthy	Prevalence 0.41
Predicted	Disease	22	0	PPV 1
Predicted	Healthy	0	32	NPV 1
		Sensitivity 1	Specificity 1	Accuracy 1

Using four proteins

Table S14: Confusion matrices when applying logistic regression to identify the samples of patients in late stage and healthy patients in data set 1 (L1 vs H1), using only CA-125 (left panel) and all four proteins (right panel). The samples from patients in late stage cancer and healthy patients in data set 1 (L1 vs H1) were used to train the model. Note that in this experiment the cohort of late stage cancer samples and healthy samples in data set 1 was divided into 60% for training and 40% for testing. This experiment is corresponding to experiment ID 4 in Table 3.

		Actual label: Disease	Actual label: Healthy	Prevalence 0.41
Predicted	Disease	17	1	PPV 0.92
Predicted	Healthy	5	31	NPV 0.94
		Sensitivity 0.92	Specificity 0.94	Accuracy 0.89

Using CA-125 alone

		Actual label: Disease	Actual label: Healthy	Prevalence 0.41
Predicted	Disease	22	0	PPV 1
Predicted	Healthy	0	32	NPV 1
		Sensitivity 1	Specificity 1	Accuracy 1

Using four proteins

Table S15: Confusion matrices when applying random forest to identify the samples of patients in late stage and healthy patients in data set 1 (L1 vs H1), using only CA-125 (left panel) and all four proteins (right panel). The samples from patients in late stage cancer and healthy patients in data set 1 (L1 vs H1) were used to train the model. Note that in this experiment the cohort of late stage cancer samples and healthy samples in data set 1 was divided into 60% for training and 40% for testing. This experiment is corresponding to experiment ID 4 in Table 3.

		Actual label: Disease	Actual label: Healthy	Prevalence 0.41
Predicted	Disease	20	2	PPV 0.91
Predicted	Healthy	2	30	NPV 0.94
		Sensitivity 0.91	Specificity 0.94	Accuracy 0.93

Using CA-125 alone

		Actual label: Disease	Actual label: Healthy	Prevalence 0.41
Predicted	Disease	22	0	PPV 1
Predicted	Healthy	0	32	NPV 1
		Sensitivity 1	Specificity 1	Accuracy 1

Using four proteins

Table S16: Confusion matrices when applying SVM to identify the samples of patients in late stage and healthy patients in data set 1 (L1 vs H1), using only CA-125 (left panel) and all four proteins (right panel). The samples from patients in late stage cancer and healthy patients in data set 1 (L1 vs H1) were used to train the model. Note that in this experiment the cohort of late stage cancer samples and healthy samples in data set 1 was divided into 60% for training and 40% for testing. This experiment is corresponding to experiment ID 4 in Table 3.

		Actual label: Disease	Actual label: Healthy	Prevalence 0.41
Predicted	Disease	17	0	PPV 1
Predicted	Healthy	5	32	NPV 0.86
		Sensitivity 0.77	Specificity 1	Accuracy 0.91

Using CA-125 alone

		Actual label: Disease	Actual label: Healthy	Prevalence 0.41
Predicted	Disease	22	0	PPV 1
Predicted	Healthy	0	32	NPV 1
		Sensitivity 1	Specificity 1	Accuracy 1

Using four proteins

Table S17: Confusion matrices when applying KNN to identify the samples of patients in late stage and healthy patients in data set 2 (L2 vs H2), using only CA-125 (left panel) and all four proteins (right panel). The samples from patients in late stage cancer and healthy patients in data set 1 (L1 vs H1) were used to train the model. This experiment is corresponding to experiment ID 5 in Table 3.

		Actual label: Disease	Actual label: Healthy	Prevalence 0.23
Predicted	Disease	40	24	PPV 0.63
Predicted	Healthy	21	175	NPV 0.89
		Sensitivity 0.66	Specificity 0.88	Accuracy 0.83

Using CA-125 alone

		Actual label: Disease	Actual label: Healthy	Prevalence 0.23
Predicted	Disease	42	5	PPV 0.89
Predicted	Healthy	19	194	NPV 0.91
		Sensitivity 0.69	Specificity 0.97	Accuracy 0.91

Using four proteins

Table S18: Confusion matrices when applying logistic regression to identify the samples of patients in late stage and healthy patients in data set 2 (L2 vs H2), using only CA-125 (left panel) and all four proteins (right panel). The samples from patients in late stage cancer and healthy patients in data set 1 (L1 vs H1) were used to train the model. This experiment is corresponding to experiment ID 5 in Table 3.

		Actual label: Disease	Actual label: Healthy	Prevalence 0.23
Predicted	Disease	40	24	PPV 0.63
Predicted	Healthy	21	175	NPV 0.89
		Sensitivity 0.66	Specificity 0.88	Accuracy 0.83

Using CA-125 alone

		Actual label: Disease	Actual label: Healthy	Prevalence 0.23
Predicted	Disease	45	5	PPV 0.9
Predicted	Healthy	16	194	NPV 0.92
		Sensitivity 0.74	Specificity 0.97	Accuracy 0.92

Using four proteins

Table S19: Confusion matrices when applying random forest to identify the samples of patients in late stage and healthy patients in data set 2 (L2 vs H2), using only CA-125 (left panel) and all four proteins (right panel). The samples from patients in late stage cancer and healthy patients in data set 1 (L1 vs H1) were used to train the model. This experiment is corresponding to experiment ID 5 in Table 3.

Predicted \ Actual label	Disease	Healthy	Prevalence 0.23
Disease	40	24	PPV 0.63
Healthy	21	175	NPV 0.89
	Sensitivity 0.66	Specificity 0.88	Accuracy 0.83

Using CA-125 alone

Predicted \ Actual label	Disease	Healthy	Prevalence 0.23
Disease	46	10	PPV 0.82
Healthy	15	189	NPV 0.93
	Sensitivity 0.75	Specificity 0.95	Accuracy 0.9

Using four proteins

Table S20: Confusion matrices when applying SVM to identify the samples of patients in late stage and healthy patients in data set 2 (L2 vs H2), using only CA-125 (left panel) and all four proteins (right panel). The samples from patients in late stage cancer and healthy patients in data set 1 (L1 vs H1) were used to train the model. This experiment is corresponding to experiment ID 5 in Table 3.

Predicted \ Actual label	Disease	Healthy	Prevalence 0.23
Disease	38	6	PPV 0.86
Healthy	23	193	NPV 0.89
	Sensitivity 0.62	Specificity 0.97	Accuracy 0.89

Using CA-125 alone

Predicted \ Actual label	Disease	Healthy	Prevalence 0.23
Disease	45	7	PPV 0.87
Healthy	16	192	NPV 0.92
	Sensitivity 0.74	Specificity 0.96	Accuracy 0.91

Using four proteins

Table S21: Confusion matrices when applying KNN to identify the samples of ovarian cancer patients and healthy patients in data set 2 (C2 vs H2), using only CA-125 (left panel) and all four proteins (right panel). The samples from patients in late stage cancer and healthy patients in data set 1 (L1 vs H1) were used to train the model. This experiment is corresponding to experiment ID 6 in Table 3.

Predicted \ Actual label	Disease	Healthy	Prevalence 0.29
Disease	53	33	PPV 0.62
Healthy	27	166	NPV 0.86
	Sensitivity 0.66	Specificity 0.83	Accuracy 0.78

Using CA-125 alone

Predicted \ Actual label	Disease	Healthy	Prevalence 0.29
Disease	56	4	PPV 0.93
Healthy	24	195	NPV 0.89
	Sensitivity 0.7	Specificity 0.98	Accuracy 0.9

Using four proteins

Table S22: Confusion matrices when applying logistic regression to identify the samples of ovarian cancer patients and healthy patients in data set 2 (C2 vs H2), using only CA-125 (left panel) and all four proteins (right panel). The samples from patients in late stage cancer and healthy patients in data set 1 (L1 vs H1) were used to train the model. This experiment is corresponding to experiment ID 6 in Table 3.

Using CA-125 alone

Predicted \ Actual label	Disease	Healthy	Prevalence 0.29
Disease	52	22	PPV 0.7
Healthy	28	177	NPV 0.86
	Sensitivity 0.65	Specificity 0.89	Accuracy 0.82

Using four proteins

Predicted \ Actual label	Disease	Healthy	Prevalence 0.29
Disease	61	7	PPV 0.9
Healthy	19	192	NPV 0.91
	Sensitivity 0.76	Specificity 0.96	Accuracy 0.91

Table S23: Confusion matrices when applying random forest to identify the samples of ovarian cancer patients and healthy patients in data set 2 (C2 vs H2), using only CA-125 (left panel) and all four proteins (right panel). The samples from patients in late stage cancer and healthy patients in data set 1 (L1 vs H1) were used to train the model. This experiment is corresponding to experiment ID 6 in Table 3.

Using CA-125 alone

Predicted \ Actual label	Disease	Healthy	Prevalence 0.29
Disease	52	32	PPV 0.62
Healthy	28	167	NPV 0.86
	Sensitivity 0.65	Specificity 0.84	Accuracy 0.78

Using four proteins

Predicted \ Actual label	Disease	Healthy	Prevalence 0.29
Disease	62	11	PPV 0.85
Healthy	18	188	NPV 0.91
	Sensitivity 0.78	Specificity 0.94	Accuracy 0.9

Table S24: Confusion matrices when applying SVM to identify the samples of ovarian cancer patients and healthy patients in data set 2 (C2 vs H2), using only CA-125 (left panel) and all four proteins (right panel). The samples from patients in late stage cancer and healthy patients in data set 1 (L1 vs H1) were used to train the model. This experiment is corresponding to experiment ID 6 in Table 3.

Using CA-125 alone

Predicted \ Actual label	Disease	Healthy	Prevalence 0.29
Disease	47	8	PPV 0.85
Healthy	33	191	NPV 0.85
	Sensitivity 0.59	Specificity 0.96	Accuracy 0.85

Using four proteins

Predicted \ Actual label	Disease	Healthy	Prevalence 0.29
Disease	61	8	PPV 0.88
Healthy	19	191	NPV 0.91
	Sensitivity 0.76	Specificity 0.96	Accuracy 0.9

Table S25: Confusion matrices when applying KNN to identify the samples of patients in early stage and healthy patients in data set 2 (E2 vs H2), using only CA-125 (left panel) and all four proteins (right panel). The samples from ovarian cancer patients and healthy patients in data set 1 (C1 vs H1) were used to train the model. This experiment is corresponding to experiment ID 7 in Table 3.

		Actual label: Disease	Actual label: Healthy	Prevalence 0.09
Predicted	Disease	15	135	PPV 0.1
Predicted	Healthy	4	64	NPV 0.94
		Sensitivity 0.79	Specificity 0.32	Accuracy 0.36

Using CA-125 alone

		Actual label: Disease	Actual label: Healthy	Prevalence 0.09
Predicted	Disease	16	46	PPV 0.26
Predicted	Healthy	3	153	NPV 0.98
		Sensitivity 0.84	Specificity 0.77	Accuracy 0.76

Using four proteins

Table S26: Confusion matrices when applying logistic regression to identify the samples of patients in early stage and healthy patients in data set 2 (E2 vs H2), using only CA-125 (left panel) and all four proteins (right panel). The samples from ovarian cancer patients and healthy patients in data set 1 (C1 vs H1) were used to train the model. This experiment is corresponding to experiment ID 7 in Table 3.

		Actual label: Disease	Actual label: Healthy	Prevalence 0.09
Predicted	Disease	14	105	PPV 0.12
Predicted	Healthy	5	94	NPV 0.95
		Sensitivity 0.74	Specificity 0.47	Accuracy 0.5

Using CA-125 alone

		Actual label: Disease	Actual label: Healthy	Prevalence 0.09
Predicted	Disease	17	53	PPV 0.24
Predicted	Healthy	2	146	NPV 0.99
		Sensitivity 0.89	Specificity 0.73	Accuracy 0.75

Using four proteins

Table S27: Confusion matrices when applying random forest to identify the samples of patients in early stage and healthy patients in data set 2 (E2 vs H2), using only CA-125 (left panel) and all four proteins (right panel). The samples from ovarian cancer patients and healthy patients in data set 1 (C1 vs H1) were used to train the model. This experiment is corresponding to experiment ID 7 in Table 3.

		Actual label: Disease	Actual label: Healthy	Prevalence 0.09
Predicted	Disease	12	89	PPV 0.12
Predicted	Healthy	7	110	NPV 0.94
		Sensitivity 0.63	Specificity 0.55	Accuracy 0.56

Using CA-125 alone

		Actual label: Disease	Actual label: Healthy	Prevalence 0.09
Predicted	Disease	17	113	PPV 0.13
Predicted	Healthy	2	86	NPV 0.98
		Sensitivity 0.89	Specificity 0.43	Accuracy 0.47

Using four proteins

Table S28: Confusion matrices when applying SVM to identify the samples of patients in early stage and healthy patients in data set 2 (E2 vs H2), using only CA-125 (left panel) and all four proteins (right panel). The samples from ovarian cancer patients and healthy patients in data set 1 (C1 vs H1) were used to train the model. This experiment is corresponding to experiment ID 7 in Table 3.

Using CA-125 alone

Predicted \ Actual label	Disease	Healthy	Prevalence 0.09
Disease	13	75	PPV 0.15
Healthy	6	124	NPV 0.95
	Sensitivity 0.68	Specificity 0.62	Accuracy 0.63

Using four proteins

Predicted \ Actual label	Disease	Healthy	Prevalence 0.09
Disease	17	108	PPV 0.14
Healthy	2	91	NPV 0.98
	Sensitivity 0.89	Specificity 0.46	Accuracy 0.5

Table S29: Confusion matrices when applying KNN to identify the samples of patients in late stage and healthy patients in data set 2 (L2 vs H2), using only CA-125 (left panel) and all four proteins (right panel). The samples from ovarian cancer patients and healthy patients in data set 1 (C1 vs H1) were used to train the model. This experiment is corresponding to experiment ID 8 in Table 3.

Using CA-125 alone

Predicted \ Actual label	Disease	Healthy	Prevalence 0.23
Disease	44	27	PPV 0.61
Healthy	17	172	NPV 0.91
	Sensitivity 0.72	Specificity 0.86	Accuracy 0.83

Using four proteins

Predicted \ Actual label	Disease	Healthy	Prevalence 0.23
Disease	42	6	PPV 0.88
Healthy	19	193	NPV 0.91
	Sensitivity 0.69	Specificity 0.97	Accuracy 0.9

Table S30: Confusion matrices when applying logistic regression to identify the samples of patients in late stage and healthy patients in data set 2 (L2 vs H2), using only CA-125 (left panel) and all four proteins (right panel). The samples from ovarian cancer patients and healthy patients in data set 1 (C1 vs H1) were used to train the model. This experiment is corresponding to experiment ID 8 in Table 3.

Using CA-125 alone

Predicted \ Actual label	Disease	Healthy	Prevalence 0.23
Disease	41	18	PPV 0.69
Healthy	20	181	NPV 0.9
	Sensitivity 0.67	Specificity 0.91	Accuracy 0.85

Using four proteins

Predicted \ Actual label	Disease	Healthy	Prevalence 0.23
Disease	45	12	PPV 0.79
Healthy	16	187	NPV 0.92
	Sensitivity 0.74	Specificity 0.94	Accuracy 0.89

Table S31: Confusion matrices when applying random forest to identify the samples of patients in late stage and healthy patients in data set 2 (L2 vs H2), using only CA-125 (left panel) and all four proteins (right panel). The samples from ovarian cancer patients and healthy patients in data set 1 (C1 vs H1) were used to train the model. This experiment is corresponding to experiment ID 8 in Table 3.

		Actual label: Disease	Actual label: Healthy	Prevalence 0.23
Predicted	Disease	41	29	PPV 0.59
Predicted	Healthy	20	170	NPV 0.89
		Sensitivity 0.67	Specificity 0.85	Accuracy 0.81

Using CA-125 alone

		Actual label: Disease	Actual label: Healthy	Prevalence 0.23
Predicted	Disease	46	11	PPV 0.81
Predicted	Healthy	15	188	NPV 0.93
		Sensitivity 0.75	Specificity 0.94	Accuracy 0.9

Using four proteins

Table S32: Confusion matrices when applying SVM to identify the samples of patients in late stage and healthy patients in data set 2 (L2 vs H2), using only CA-125 (left panel) and all four proteins (right panel). The samples from ovarian cancer patients and healthy patients in data set 1 (C1 vs H1) were used to train the model. This experiment is corresponding to experiment ID 8 in Table 3.

		Actual label: Disease	Actual label: Healthy	Prevalence 0.23
Predicted	Disease	39	12	PPV 0.76
Predicted	Healthy	22	187	NPV 0.89
		Sensitivity 0.64	Specificity 0.94	Accuracy 0.87

Using CA-125 alone

		Actual label: Disease	Actual label: Healthy	Prevalence 0.23
Predicted	Disease	45	11	PPV 0.8
Predicted	Healthy	16	188	NPV 0.92
		Sensitivity 0.74	Specificity 0.94	Accuracy 0.89

Using four proteins

Table S33: Confusion matrices when applying KNN to identify the samples of ovarian cancer patients and healthy patients in data set 2 (C2 vs H2), using only CA-125 (left panel) and all four proteins (right panel). The samples from ovarian cancer patients and healthy patients in data set 1 (C1 vs H1) were used to train the model. This experiment is corresponding to experiment ID 9 in Table 3.

		Actual label: Disease	Actual label: Healthy	Prevalence 0.29
Predicted	Disease	59	35	PPV 0.63
Predicted	Healthy	21	164	NPV 0.89
		Sensitivity 0.74	Specificity 0.82	Accuracy 0.8

Using CA-125 alone

		Actual label: Disease	Actual label: Healthy	Prevalence 0.29
Predicted	Disease	58	13	PPV 0.82
Predicted	Healthy	22	186	NPV 0.89
		Sensitivity 0.73	Specificity 0.93	Accuracy 0.87

Using four proteins

Table S34: Confusion matrices when applying logistic regression to identify the samples of ovarian cancer patients and healthy patients in data set 2 (C2 vs H2), using only CA-125 (left panel) and all four proteins (right panel). The samples from ovarian cancer patients and healthy patients in data set 1 (C1 vs H1) were used to train the model. This experiment is corresponding to experiment ID 9 in Table 3.

Using CA-125 alone

Predicted \ Actual label	Disease	Healthy	Prevalence 0.29
Disease	56	27	PPV 0.67
Healthy	24	172	NPV 0.88
	Sensitivity 0.7	Specificity 0.86	Accuracy 0.82

Using four proteins

Predicted \ Actual label	Disease	Healthy	Prevalence 0.29
Disease	61	12	PPV 0.84
Healthy	19	187	NPV 0.91
	Sensitivity 0.76	Specificity 0.94	Accuracy 0.89

Table S35: Confusion matrices when applying random forest to identify the samples of ovarian cancer patients and healthy patients in data set 2 (C2 vs H2), using only CA-125 (left panel) and all four proteins (right panel). The samples from ovarian cancer patients and healthy patients in data set 1 (C1 vs H1) were used to train the model. This experiment is corresponding to experiment ID 9 in Table 3.

Using CA-125 alone

Predicted \ Actual label	Disease	Healthy	Prevalence 0.29
Disease	55	41	PPV 0.57
Healthy	25	158	NPV 0.86
	Sensitivity 0.69	Specificity 0.79	Accuracy 0.76

Using four proteins

Predicted \ Actual label	Disease	Healthy	Prevalence 0.29
Disease	62	13	PPV 0.83
Healthy	18	186	NPV 0.91
	Sensitivity 0.78	Specificity 0.93	Accuracy 0.89

Table S36: Confusion matrices when applying SVM to identify the samples of ovarian cancer patients and healthy patients in data set 2 (C2 vs H2), using only CA-125 (left panel) and all four proteins (right panel). The samples from ovarian cancer patients and healthy patients in data set 1 (C1 vs H1) were used to train the model. This experiment is corresponding to experiment ID 9 in Table 3.

Using CA-125 alone

Predicted \ Actual label	Disease	Healthy	Prevalence 0.29
Disease	51	14	PPV 0.78
Healthy	29	185	NPV 0.86
	Sensitivity 0.64	Specificity 0.93	Accuracy 0.85

Using four proteins

Predicted \ Actual label	Disease	Healthy	Prevalence 0.29
Disease	62	11	PPV 0.85
Healthy	18	188	NPV 0.91
	Sensitivity 0.76	Specificity 0.94	Accuracy 0.9

Table S37: Confusion matrices when applying KNN to identify the samples of patients in early stage and healthy patients in data set 2 (E2 vs H2), using only CA-125 (left panel) and all four proteins (right panel). The samples from patients in early stage cancer and healthy patients in data set 2 (E2 vs E2) were used to train the model. Note that in this experiment the cohort of early stage cancer samples and healthy samples in data set 2 was divided into 60% for training and 40% for testing. This experiment is corresponding to experiment ID 9 in Table 3. This experiment is corresponding to experiment ID 10 in Table 3.

Using CA-125 alone

	Actual label: Disease	Actual label: Healthy	Prevalence 0.08
Predicted: Disease	1	2	PPV 0.33
Predicted: Healthy	6	77	NPV 0.93
	Sensitivity 0.14	Specificity 0.97	Accuracy 0.91

Using four proteins

	Actual label: Disease	Actual label: Healthy	Prevalence 0.08
Predicted: Disease	2	1	PPV 0.67
Predicted: Healthy	5	78	NPV 0.94
	Sensitivity 0.29	Specificity 0.99	Accuracy 0.93

Table S38: Confusion matrices when applying logistic regression to identify the samples of patients in early stage and healthy patients in data set 2 (E2 vs H2), using only CA-125 (left panel) and all four proteins (right panel). The samples from patients in early stage cancer and healthy patients in data set 2 (E2 vs E2) were used to train the model. Note that in this experiment the cohort of early stage cancer samples and healthy samples in data set 2 was divided into 60% for training and 40% for testing. This experiment is corresponding to experiment ID 10 in Table 3.

Using CA-125 alone

	Actual label: Disease	Actual label: Healthy	Prevalence 0.08
Predicted: Disease	1	2	PPV 0.33
Predicted: Healthy	6	77	NPV 0.93
	Sensitivity 0.14	Specificity 0.97	Accuracy 0.91

Using four proteins

	Actual label: Disease	Actual label: Healthy	Prevalence 0.08
Predicted: Disease	4	1	PPV 0.8
Predicted: Healthy	3	78	NPV 0.96
	Sensitivity 0.57	Specificity 0.99	Accuracy 0.95

Table S39: Confusion matrices when applying random forest to identify the samples of patients in early stage and healthy patients in data set 2 (E2 vs H2), using only CA-125 (left panel) and all four proteins (right panel). The samples from patients in early stage cancer and healthy patients in data set 2 (E2 vs E2) were used to train the model. Note that in this experiment the cohort of early stage cancer samples and healthy samples in data set 2 was divided into 60% for training and 40% for testing. This experiment is corresponding to experiment ID 10 in Table 3.

Using CA-125 alone

	Actual label: Disease	Actual label: Healthy	Prevalence 0.08
Predicted: Disease	1	5	PPV 0.17
Predicted: Healthy	6	74	NPV 0.93
	Sensitivity 0.14	Specificity 0.94	Accuracy 0.87

Using four proteins

	Actual label: Disease	Actual label: Healthy	Prevalence 0.08
Predicted: Disease	3	2	PPV 0.6
Predicted: Healthy	4	77	NPV 0.95
	Sensitivity 0.43	Specificity 0.97	Accuracy 0.93

Table S40: Confusion matrices when applying SVM to identify the samples of patients in early stage and healthy patients in data set 2 (E2 vs H2), using only CA-125 (left panel) and all four proteins (right panel). The samples from patients in early stage cancer and healthy patients in data set 2 (E2 vs E2) were used to train the model. Note that in this experiment the cohort of early stage cancer samples and healthy samples in data set 2 was divided into 60% for training and 40% for testing. This experiment is corresponding to experiment ID 10 in Table 3.

Using CA-125 alone

Predicted \ Actual label	Disease	Healthy	Prevalence 0.08
Disease	1	2	PPV 0.33
Healthy	6	77	NPV 0.93
	Sensitivity 0.14	Specificity 0.97	Accuracy 0.91

Using four proteins

Predicted \ Actual label	Disease	Healthy	Prevalence 0.08
Disease	2	1	PPV 0.67
Healthy	5	78	NPV 0.94
	Sensitivity 0.29	Specificity 0.99	Accuracy 0.93

Table S41: Confusion matrices when applying KNN to identify the samples of patients in early stage and healthy patients in data set 1 (E1 vs H1), using only CA-125 (left panel) and all four proteins (right panel). The samples from patients in early stage cancer and healthy patients in data set 2 (E2 vs E2) were used to train the model. This experiment is corresponding to experiment ID 11 in Table 3.

Using CA-125 alone

Predicted \ Actual label	Disease	Healthy	Prevalence 0.18
Disease	5	1	PPV 0.83
Healthy	13	79	NPV 0.86
	Sensitivity 0.28	Specificity 0.99	Accuracy 0.86

Using four proteins

Predicted \ Actual label	Disease	Healthy	Prevalence 0.18
Disease	12	0	PPV 1
Healthy	6	80	NPV 0.93
	Sensitivity 0.67	Specificity 1	Accuracy 0.94

Table S42: Confusion matrices when applying logistic regression to identify the samples of patients in early stage and healthy patients in data set 1 (E1 vs H1), using only CA-125 (left panel) and all four proteins (right panel). The samples from patients in early stage cancer and healthy patients in data set 2 (E2 vs E2) were used to train the model. This experiment is corresponding to experiment ID 11 in Table 3.

Using CA-125 alone

Predicted \ Actual label	Disease	Healthy	Prevalence 0.18
Disease	4	0	PPV 1
Healthy	14	80	NPV 0.85
	Sensitivity 0.22	Specificity 1	Accuracy 0.86

Using four proteins

Predicted \ Actual label	Disease	Healthy	Prevalence 0.18
Disease	15	0	PPV 1
Healthy	3	80	NPV 0.96
	Sensitivity 0.83	Specificity 1	Accuracy 0.97

Table S43: Confusion matrices when applying random forest to identify the samples of patients in early stage and healthy patients in data set 1 (E1 vs H1), using only CA-125 (left panel) and all four proteins (right panel). The samples from patients in early stage cancer and healthy patients in data set 2 (E2 vs E2) were used to train the model. This experiment is corresponding to experiment ID 11 in Table 3.

Predicted \ Actual label	Disease	Healthy	Prevalence 0.18
Disease	6	4	PPV 0.6
Healthy	12	76	NPV 0.86
	Sensitivity 0.33	Specificity 0.95	Accuracy 0.84

Using CA-125 alone

Predicted \ Actual label	Disease	Healthy	Prevalence 0.18
Disease	16	0	PPV 1
Healthy	2	80	NPV 0.98
	Sensitivity 0.89	Specificity 1	Accuracy 0.98

Using four proteins

Table S44: Confusion matrices when applying SVM to identify the samples of patients in early stage and healthy patients in data set 1 (E1 vs H1), using only CA-125 (left panel) and all four proteins (right panel). The samples from patients in early stage cancer and healthy patients in data set 2 (E2 vs E2) were used to train the model. This experiment is corresponding to experiment ID 11 in Table 3.

Predicted \ Actual label	Disease	Healthy	Prevalence 0.18
Disease	4	0	PPV 1
Healthy	14	80	NPV 0.85
	Sensitivity 0.22	Specificity 1	Accuracy 0.86

Using CA-125 alone

Predicted \ Actual label	Disease	Healthy	Prevalence 0.18
Disease	15	0	PPV 1
Healthy	3	80	NPV 0.96
	Sensitivity 0.83	Specificity 1	Accuracy 0.97

Using four proteins

Table S45: Confusion matrices when applying KNN to identify the samples of ovarian cancer patients and healthy patients in data set 1 (C1 vs H1), using only CA-125 (left panel) and all four proteins (right panel). The samples from patients in early stage cancer and healthy patients in data set 2 (E2 vs E2) were used to train the model. This experiment is corresponding to experiment ID 12 in Table 3.

Predicted \ Actual label	Disease	Healthy	Prevalence 0.48
Disease	28	1	PPV 0.97
Healthy	45	79	NPV 0.64
	Sensitivity 0.38	Specificity 0.99	Accuracy 0.7

Using CA-125 alone

Predicted \ Actual label	Disease	Healthy	Prevalence 0.48
Disease	24	0	PPV 1
Healthy	49	80	NPV 0.62
	Sensitivity 0.33	Specificity 1	Accuracy 0.68

Using four proteins

Table S46: Confusion matrices when applying logistic regression to identify the samples of ovarian cancer patients and healthy patients in data set 1 (C1 vs H1), using only CA-125 (left panel) and all four proteins (right panel). The samples from patients in early stage cancer and healthy patients in data set 2 (E2 vs E2) were used to train the model. This experiment is corresponding to experiment ID 12 in Table 3.

	Actual label: Disease	Actual label: Healthy	Prevalence 0.48
Predicted: Disease	26	0	PPV 1
Predicted: Healthy	47	80	NPV 0.63
	Sensitivity 0.36	Specificity 1	Accuracy 0.69

Using CA-125 alone

	Actual label: Disease	Actual label: Healthy	Prevalence 0.48
Predicted: Disease	38	0	PPV 1
Predicted: Healthy	35	80	NPV 0.7
	Sensitivity 0.52	Specificity 1	Accuracy 0.77

Using four proteins

Table S47: Confusion matrices when applying random forest to identify the samples of ovarian cancer patients and healthy patients in data set 1 (C1 vs H1), using only CA-125 (left panel) and all four proteins (right panel). The samples from patients in early stage cancer and healthy patients in data set 2 (E2 vs E2) were used to train the model. This experiment is corresponding to experiment ID 12 in Table 3.

	Actual label: Disease	Actual label: Healthy	Prevalence 0.48
Predicted: Disease	31	3	PPV 0.91
Predicted: Healthy	42	77	NPV 0.65
	Sensitivity 0.42	Specificity 0.96	Accuracy 0.71

Using CA-125 alone

	Actual label: Disease	Actual label: Healthy	Prevalence 0.48
Predicted: Disease	42	0	PPV 1
Predicted: Healthy	31	80	NPV 0.72
	Sensitivity 0.58	Specificity 1	Accuracy 0.8

Using four proteins

Table S48: Confusion matrices when applying SVM to identify the samples of ovarian cancer patients and healthy patients in data set 1 (C1 vs H1), using only CA-125 (left panel) and all four proteins (right panel). The samples from patients in early stage cancer and healthy patients in data set 2 (E2 vs E2) were used to train the model. This experiment is corresponding to experiment ID 12 in Table 3.

	Actual label: Disease	Actual label: Healthy	Prevalence 0.48
Predicted: Disease	27	0	PPV 1
Predicted: Healthy	46	80	NPV 0.63
	Sensitivity 0.37	Specificity 1	Accuracy 0.7

Using CA-125 alone

	Actual label: Disease	Actual label: Healthy	Prevalence 0.48
Predicted: Disease	50	0	PPV 1
Predicted: Healthy	23	80	NPV 0.78
	Sensitivity 0.68	Specificity 1	Accuracy 0.85

Using four proteins

Table S49: Confusion matrices when applying KNN to identify the samples of patients in late stage and healthy patients in data set 2 (L2 vs H2), using only CA-125 (left panel) and all four proteins (right panel). The samples from patients in late stage cancer and healthy patients in data set 2 (L2 vs L2) were used to train the model. Note that in this experiment the cohort of late stage cancer samples and healthy samples in data set 2 was divided into 60% for training and 40% for testing. This experiment is corresponding to experiment ID 13 in Table 3.

Actual label

Predicted	Disease	Healthy	Prevalence 0.23
Disease	13	2	PPV 0.87
Healthy	11	77	NPV 0.88
	Sensitivity 0.54	Specificity 0.97	Accuracy 0.87

Using CA-125 alone

Actual label

Predicted	Disease	Healthy	Prevalence 0.23
Disease	16	1	PPV 0.94
Healthy	8	78	NPV 0.91
	Sensitivity 0.67	Specificity 0.98	Accuracy 0.91

Using four proteins

Table S50: Confusion matrices when applying logistic regression to identify the samples of patients in late stage and healthy patients in data set 2 (L2 vs H2), using only CA-125 (left panel) and all four proteins (right panel). The samples from patients in late stage cancer and healthy patients in data set 2 (L2 vs L2) were used to train the model. Note that in this experiment the cohort of late stage cancer samples and healthy samples in data set 2 was divided into 60% for training and 40% for testing. This experiment is corresponding to experiment ID 13 in Table 3.

Actual label

Predicted	Disease	Healthy	Prevalence 0.23
Disease	11	2	PPV 0.85
Healthy	13	77	NPV 0.86
	Sensitivity 0.46	Specificity 0.97	Accuracy 0.85

Using CA-125 alone

Actual label

Predicted	Disease	Healthy	Prevalence 0.23
Disease	16	2	PPV 0.89
Healthy	8	77	NPV 0.91
	Sensitivity 0.67	Specificity 0.97	Accuracy 0.9

Using four proteins

Table S51: Confusion matrices when applying random forest to identify the samples of patients in late stage and healthy patients in data set 2 (L2 vs H2), using only CA-125 (left panel) and all four proteins (right panel). The samples from patients in late stage cancer and healthy patients in data set 2 (L2 vs L2) were used to train the model. Note that in this experiment the cohort of late stage cancer samples and healthy samples in data set 2 was divided into 60% for training and 40% for testing. This experiment is corresponding to experiment ID 13 in Table 3.

Actual label

Predicted	Disease	Healthy	Prevalence 0.23
Disease	14	10	PPV 0.58
Healthy	10	69	NPV 0.87
	Sensitivity 0.58	Specificity 0.87	Accuracy 0.81

Using CA-125 alone

Actual label

Predicted	Disease	Healthy	Prevalence 0.23
Disease	17	3	PPV 0.85
Healthy	7	76	NPV 0.92
	Sensitivity 0.71	Specificity 0.96	Accuracy 0.9

Using four proteins

Table S52: Confusion matrices when applying SVM to identify the samples of patients in late stage and healthy patients in data set 2 (L2 vs H2), using only CA-125 (left panel) and all four proteins (right panel). The samples from patients in late stage cancer and healthy patients in data set 2 (L2 vs L2) were used to train the model. Note that in this experiment the cohort of late stage cancer samples and healthy samples in data set 2 was divided into 60% for training and 40% for testing. This experiment is corresponding to experiment ID 13 in Table 3.

Using CA-125 alone

		Actual label: Disease	Actual label: Healthy	Prevalence 0.23
Predicted	Disease	11	2	PPV 0.85
Predicted	Healthy	13	77	NPV 0.86
		Sensitivity 0.46	Specificity 0.97	Accuracy 0.85

Using four proteins

		Actual label: Disease	Actual label: Healthy	Prevalence 0.23
Predicted	Disease	16	1	PPV 0.94
Predicted	Healthy	8	78	NPV 0.91
		Sensitivity 0.67	Specificity 0.99	Accuracy 0.91

Table S53: Confusion matrices when applying KNN to identify the samples of patients in late stage and healthy patients in data set 1 (L1 vs H1), using only CA-125 (left panel) and all four proteins (right panel). The samples from patients in late stage cancer and healthy patients in data set 2 (L2 vs L2) were used to train the model. This experiment is corresponding to experiment ID 14 in Table 3.

Using CA-125 alone

		Actual label: Disease	Actual label: Healthy	Prevalence 0.41
Predicted	Disease	43	0	PPV 1
Predicted	Healthy	12	80	NPV 0.87
		Sensitivity 0.78	Specificity 1	Accuracy 0.91

Using four proteins

		Actual label: Disease	Actual label: Healthy	Prevalence 0.41
Predicted	Disease	49	1	PPV 0.98
Predicted	Healthy	6	79	NPV 0.93
		Sensitivity 0.89	Specificity 0.99	Accuracy 0.95

Table S54: Confusion matrices when applying logistic regression to identify the samples of patients in late stage and healthy patients in data set 1 (L1 vs H1), using only CA-125 (left panel) and all four proteins (right panel). The samples from patients in late stage cancer and healthy patients in data set 2 (L2 vs L2) were used to train the model. This experiment is corresponding to experiment ID 14 in Table 3.

Using CA-125 alone

		Actual label: Disease	Actual label: Healthy	Prevalence 0.41
Predicted	Disease	42	0	PPV 1
Predicted	Healthy	13	80	NPV 0.86
		Sensitivity 0.76	Specificity 1	Accuracy 0.9

Using four proteins

		Actual label: Disease	Actual label: Healthy	Prevalence 0.41
Predicted	Disease	48	0	PPV 1
Predicted	Healthy	7	80	NPV 0.92
		Sensitivity 0.87	Specificity 1	Accuracy 0.95

Table S55: Confusion matrices when applying random forest to identify the samples of patients in late stage and healthy patients in data set 1 (L1 vs H1), using only CA-125 (left panel) and all four proteins (right panel). The samples from patients in late stage cancer and healthy patients in data set 2 (L2 vs L2) were used to train the model. This experiment is corresponding to experiment ID 14 in Table 3.

		Actual label		
		Disease	Healthy	Prevalence 0.41
Predicted	Disease	42	11	PPV 0.79
Predicted	Healthy	13	69	NPV 0.84
		Sensitivity 0.76	Specificity 0.86	Accuracy 0.82

Using CA-125 alone

		Actual label		
		Disease	Healthy	Prevalence 0.41
Predicted	Disease	52	9	PPV 0.85
Predicted	Healthy	3	71	NPV 0.96
		Sensitivity 0.95	Specificity 0.89	Accuracy 0.91

Using four proteins

Table S56: Confusion matrices when applying SVM to identify the samples of patients in late stage and healthy patients in data set 1 (L1 vs H1), using only CA-125 (left panel) and all four proteins (right panel). The samples from patients in late stage cancer and healthy patients in data set 2 (L2 vs L2) were used to train the model. This experiment is corresponding to experiment ID 14 in Table 3.

		Actual label		
		Disease	Healthy	Prevalence 0.41
Predicted	Disease	42	0	PPV 1
Predicted	Healthy	13	80	NPV 0.86
		Sensitivity 0.76	Specificity 1	Accuracy 0.9

Using CA-125 alone

		Actual label		
		Disease	Healthy	Prevalence 0.41
Predicted	Disease	48	0	PPV 1
Predicted	Healthy	7	80	NPV 0.92
		Sensitivity 0.87	Specificity 1	Accuracy 0.95

Using four proteins

Table S57: Confusion matrices when applying KNN to identify the samples of ovarian cancer patients and healthy patients in data set 1 (C1 vs H1), using only CA-125 (left panel) and all four proteins (right panel). The samples from patients in late stage cancer and healthy patients in data set 2 (L2 vs L2) were used to train the model. This experiment is corresponding to experiment ID 15 in Table 3.

		Actual label		
		Disease	Healthy	Prevalence 0.48
Predicted	Disease	53	0	PPV 1
Predicted	Healthy	20	80	NPV 0.8
		Sensitivity 0.73	Specificity 1	Accuracy 0.87

Using CA-125 alone

		Actual label		
		Disease	Healthy	Prevalence 0.48
Predicted	Disease	65	4	PPV 0.94
Predicted	Healthy	8	76	NPV 0.9
		Sensitivity 0.89	Specificity 0.95	Accuracy 0.92

Using four proteins

Table S58: Confusion matrices when applying logistic regression to identify the samples of ovarian cancer patients and healthy patients in data set 1 (C1 vs H1), using only CA-125 (left panel) and all four proteins (right panel). The samples from patients in late stage cancer and healthy patients in data set 2 (L2 vs L2) were used to train the model. This experiment is corresponding to experiment ID 15 in Table 3.

Using CA-125 alone

Predicted \ Actual label	Disease	Healthy	Prevalence 0.48
Disease	51	0	PPV 1
Healthy	22	80	NPV 0.78
	Sensitivity 0.7	Specificity 1	Accuracy 0.86

Using four proteins

Predicted \ Actual label	Disease	Healthy	Prevalence 0.48
Disease	59	0	PPV 1
Healthy	14	80	NPV 0.85
	Sensitivity 0.81	Specificity 1	Accuracy 0.91

Table S59: Confusion matrices when applying random forest to identify the samples of ovarian cancer patients and healthy patients in data set 1 (C1 vs H1), using only CA-125 (left panel) and all four proteins (right panel). The samples from patients in late stage cancer and healthy patients in data set 2 (L2 vs L2) were used to train the model. This experiment is corresponding to experiment ID 15 in Table 3.

Using CA-125 alone

Predicted \ Actual label	Disease	Healthy	Prevalence 0.48
Disease	52	11	PPV 0.83
Healthy	21	69	NPV 0.77
	Sensitivity 0.71	Specificity 0.86	Accuracy 0.79

Using four proteins

Predicted \ Actual label	Disease	Healthy	Prevalence 0.48
Disease	68	10	PPV 0.87
Healthy	5	70	NPV 0.93
	Sensitivity 0.93	Specificity 0.88	Accuracy 0.9

Table S60: Confusion matrices when applying SVM to identify the samples of ovarian cancer patients and healthy patients in data set 1 (C1 vs H1), using only CA-125 (left panel) and all four proteins (right panel). The samples from patients in late stage cancer and healthy patients in data set 2 (L2 vs L2) were used to train the model. This experiment is corresponding to experiment ID 15 in Table 3.

Using CA-125 alone

Predicted \ Actual label	Disease	Healthy	Prevalence 0.48
Disease	50	0	PPV 1
Healthy	23	80	NPV 0.78
	Sensitivity 0.68	Specificity 1	Accuracy 0.85

Using four proteins

Predicted \ Actual label	Disease	Healthy	Prevalence 0.48
Disease	63	0	PPV 1
Healthy	10	80	NPV 0.89
	Sensitivity 0.86	Specificity 1	Accuracy 0.93

Table S61: Confusion matrices when applying KNN to identify the samples of patients in early stage and healthy patients in data set 1 (E1 vs H1), using only CA-125 (left panel) and all four proteins (right panel). The samples from ovarian cancer patients and healthy patients in data set 2 (C2 vs H2) were used to train the model. This experiment is corresponding to experiment ID 16 in Table 3.

Using CA-125 alone

		Actual label		
		Disease	Healthy	Prevalence 0.18
Predicted	Disease	12	2	PPV 0.86
Predicted	Healthy	6	78	NPV 0.93
		Sensitivity 0.67	Specificity 0.85	Accuracy 0.93

Using four proteins

		Actual label		
		Disease	Healthy	Prevalence 0.18
Predicted	Disease	17	12	PPV 0.59
Predicted	Healthy	1	68	NPV 0.99
		Sensitivity 0.94	Specificity 0.85	Accuracy 0.87

Table S62: Confusion matrices when applying logistic regression to identify the samples of patients in early stage and healthy patients in data set 1 (E1 vs H1), using only CA-125 (left panel) and all four proteins (right panel). The samples from ovarian cancer patients and healthy patients in data set 2 (C2 vs H2) were used to train the model. This experiment is corresponding to experiment ID 16 in Table 3.

Using CA-125 alone

		Actual label		
		Disease	Healthy	Prevalence 0.18
Predicted	Disease	11	1	PPV 0.92
Predicted	Healthy	7	79	NPV 0.92
		Sensitivity 0.61	Specificity 0.99	Accuracy 0.92

Using four proteins

		Actual label		
		Disease	Healthy	Prevalence 0.18
Predicted	Disease	17	7	PPV 0.71
Predicted	Healthy	1	73	NPV 0.99
		Sensitivity 0.94	Specificity 0.91	Accuracy 0.92

Table S63: Confusion matrices when applying random forest to identify the samples of patients in early stage and healthy patients in data set 1 (E1 vs H1), using only CA-125 (left panel) and all four proteins (right panel). The samples from ovarian cancer patients and healthy patients in data set 2 (C2 vs H2) were used to train the model. This experiment is corresponding to experiment ID 16 in Table 3.

Using CA-125 alone

		Actual label		
		Disease	Healthy	Prevalence 0.18
Predicted	Disease	13	19	PPV 0.41
Predicted	Healthy	5	61	NPV 0.92
		Sensitivity 0.72	Specificity 0.76	Accuracy 0.76

Using four proteins

		Actual label		
		Disease	Healthy	Prevalence 0.18
Predicted	Disease	17	35	PPV 0.33
Predicted	Healthy	1	45	NPV 0.98
		Sensitivity 0.94	Specificity 0.56	Accuracy 0.63

Table S64: Confusion matrices when applying SVM to identify the samples of patients in early stage and healthy patients in data set 1 (E1 vs H1), using only CA-125 (left panel) and all four proteins (right panel). The samples from ovarian cancer patients and healthy patients in data set 2 (C2 vs H2) were used to train the model. This experiment is corresponding to experiment ID 16 in Table 3.

	Actual label: Disease	Healthy	Prevalence 0.18
Predicted: Disease	10	0	PPV 1
Predicted: Healthy	8	80	NPV 0.91
	Sensitivity 0.56	Specificity 1	Accuracy 0.92

Using CA-125 alone

	Actual label: Disease	Healthy	Prevalence 0.18
Predicted: Disease	17	9	PPV 0.65
Predicted: Healthy	1	71	NPV 0.99
	Sensitivity 0.94	Specificity 0.89	Accuracy 0.9

Using four proteins

Table S65: Confusion matrices when applying KNN to identify the samples of patients in late stage and healthy patients in data set 1 (L1 vs H1), using only CA-125 (left panel) and all four proteins (right panel). The samples from ovarian cancer patients and healthy patients in data set 2 (C2 vs H2) were used to train the model. This experiment is corresponding to experiment ID 17 in Table 3.

	Actual label: Disease	Healthy	Prevalence 0.41
Predicted: Disease	42	0	PPV 1
Predicted: Healthy	13	80	NPV 0.86
	Sensitivity 0.76	Specificity 1	Accuracy 0.9

Using CA-125 alone

	Actual label: Disease	Healthy	Prevalence 0.41
Predicted: Disease	48	2	PPV 0.96
Predicted: Healthy	7	78	NPV 0.92
	Sensitivity 0.87	Specificity 0.98	Accuracy 0.93

Using four proteins

Table S66: Confusion matrices when applying logistic regression to identify the samples of patients in late stage and healthy patients in data set 1 (L1 vs H1), using only CA-125 (left panel) and all four proteins (right panel). The samples from ovarian cancer patients and healthy patients in data set 2 (C2 vs H2) were used to train the model. This experiment is corresponding to experiment ID 17 in Table 3.

	Actual label: Disease	Healthy	Prevalence 0.41
Predicted: Disease	42	0	PPV 1
Predicted: Healthy	13	80	NPV 0.86
	Sensitivity 0.76	Specificity 1	Accuracy 0.9

Using CA-125 alone

	Actual label: Disease	Healthy	Prevalence 0.41
Predicted: Disease	48	0	PPV 1
Predicted: Healthy	7	80	NPV 0.92
	Sensitivity 0.87	Specificity 1	Accuracy 0.95

Using four proteins

Table S67: Confusion matrices when applying random forest to identify the samples of patients in late stage and healthy patients in data set 1 (L1 vs H1), using only CA-125 (left panel) and all four proteins (right panel). The samples from ovarian cancer patients and healthy patients in data set 2 (C2 vs H2) were used to train the model. This experiment is corresponding to experiment ID 17 in Table 3.

Using CA-125 alone

		Actual label: Disease	Actual label: Healthy	Prevalence 0.41
Predicted	Disease	43	10	PPV 0.81
Predicted	Healthy	12	70	NPV 0.85
		Sensitivity 0.78	Specificity 0.88	Accuracy 0.83

Using four proteins

		Actual label: Disease	Actual label: Healthy	Prevalence 0.41
Predicted	Disease	51	7	PPV 0.88
Predicted	Healthy	4	73	NPV 0.95
		Sensitivity 0.93	Specificity 0.91	Accuracy 0.92

Table S68: Confusion matrices when applying SVM to identify the samples of patients in late stage and healthy patients in data set 1 (L1 vs H1), using only CA-125 (left panel) and all four proteins (right panel). The samples from ovarian cancer patients and healthy patients in data set 2 (C2 vs H2) were used to train the model. This experiment is corresponding to experiment ID 17 in Table 3.

Using CA-125 alone

		Actual label: Disease	Actual label: Healthy	Prevalence 0.41
Predicted	Disease	41	0	PPV 1
Predicted	Healthy	14	80	NPV 0.85
		Sensitivity 0.75	Specificity 1	Accuracy 0.9

Using four proteins

		Actual label: Disease	Actual label: Healthy	Prevalence 0.41
Predicted	Disease	48	0	PPV 1
Predicted	Healthy	7	80	NPV 0.92
		Sensitivity 0.87	Specificity 1	Accuracy 0.95

Table S69: Confusion matrices when applying KNN to identify the samples of ovarian cancer patients and healthy patients in data set 1 (C1 vs H1), using only CA-125 (left panel) and all four proteins (right panel). The samples from ovarian cancer patients and healthy patients in data set 2 (C2 vs H2) were used to train the model. This experiment is corresponding to experiment ID 18 in Table 3.

Using CA-125 alone

		Actual label: Disease	Actual label: Healthy	Prevalence 0.48
Predicted	Disease	52	0	PPV 1
Predicted	Healthy	21	80	NPV 0.78
		Sensitivity 0.71	Specificity 1	Accuracy 0.86

Using four proteins

		Actual label: Disease	Actual label: Healthy	Prevalence 0.48
Predicted	Disease	65	0	PPV 1
Predicted	Healthy	8	80	NPV 0.91
		Sensitivity 0.89	Specificity 1	Accuracy 0.95

Table S70: Confusion matrices when applying logistic regression to identify the samples of ovarian cancer patients and healthy patients in data set 1 (C1 vs H1), using only CA-125 (left panel) and all four proteins (right panel). The samples from ovarian cancer patients and healthy patients in data set 2 (C2 vs H2) were used to train the model. This experiment is corresponding to experiment ID 18 in Table 3.

		Actual label		
		Disease	Healthy	Prevalence 0.48
Predicted	Disease	51	0	PPV 1
	Healthy	22	80	NPV 0.78
		Sensitivity 0.7	Specificity 1	Accuracy 0.86

Using CA-125 alone

		Actual label		
		Disease	Healthy	Prevalence 0.48
Predicted	Disease	66	0	PPV 1
	Healthy	7	80	NPV 0.92
		Sensitivity 0.9	Specificity 1	Accuracy 0.95

Using four proteins

Table S71: Confusion matrices when applying random forest to identify the samples of ovarian cancer patients and healthy patients in data set 1 (C1 vs H1), using only CA-125 (left panel) and all four proteins (right panel). The samples from ovarian cancer patients and healthy patients in data set 2 (C2 vs H2) were used to train the model. This experiment is corresponding to experiment ID 18 in Table 3.

		Actual label		
		Disease	Healthy	Prevalence 0.48
Predicted	Disease	56	10	PPV 0.85
	Healthy	17	70	NPV 0.8
		Sensitivity 0.77	Specificity 0.88	Accuracy 0.82

Using CA-125 alone

		Actual label		
		Disease	Healthy	Prevalence 0.48
Predicted	Disease	67	10	PPV 0.87
	Healthy	6	70	NPV 0.92
		Sensitivity 0.92	Specificity 0.88	Accuracy 0.9

Using four proteins

Table S72: Confusion matrices when applying SVM to identify the samples of ovarian cancer patients and healthy patients in data set 1 (C1 vs H1), using only CA-125 (left panel) and all four proteins (right panel). The samples from ovarian cancer patients and healthy patients in data set 2 (C2 vs H2) were used to train the model. This experiment is corresponding to experiment ID 18 in Table 3.

		Actual label		
		Disease	Healthy	Prevalence 0.48
Predicted	Disease	49	0	PPV 1
	Healthy	24	80	NPV 0.77
		Sensitivity 0.67	Specificity 1	Accuracy 0.84

Using CA-125 alone

		Actual label		
		Disease	Healthy	Prevalence 0.48
Predicted	Disease	64	0	PPV 1
	Healthy	9	80	NPV 0.9
		Sensitivity 0.88	Specificity 1	Accuracy 0.94

Using four proteins

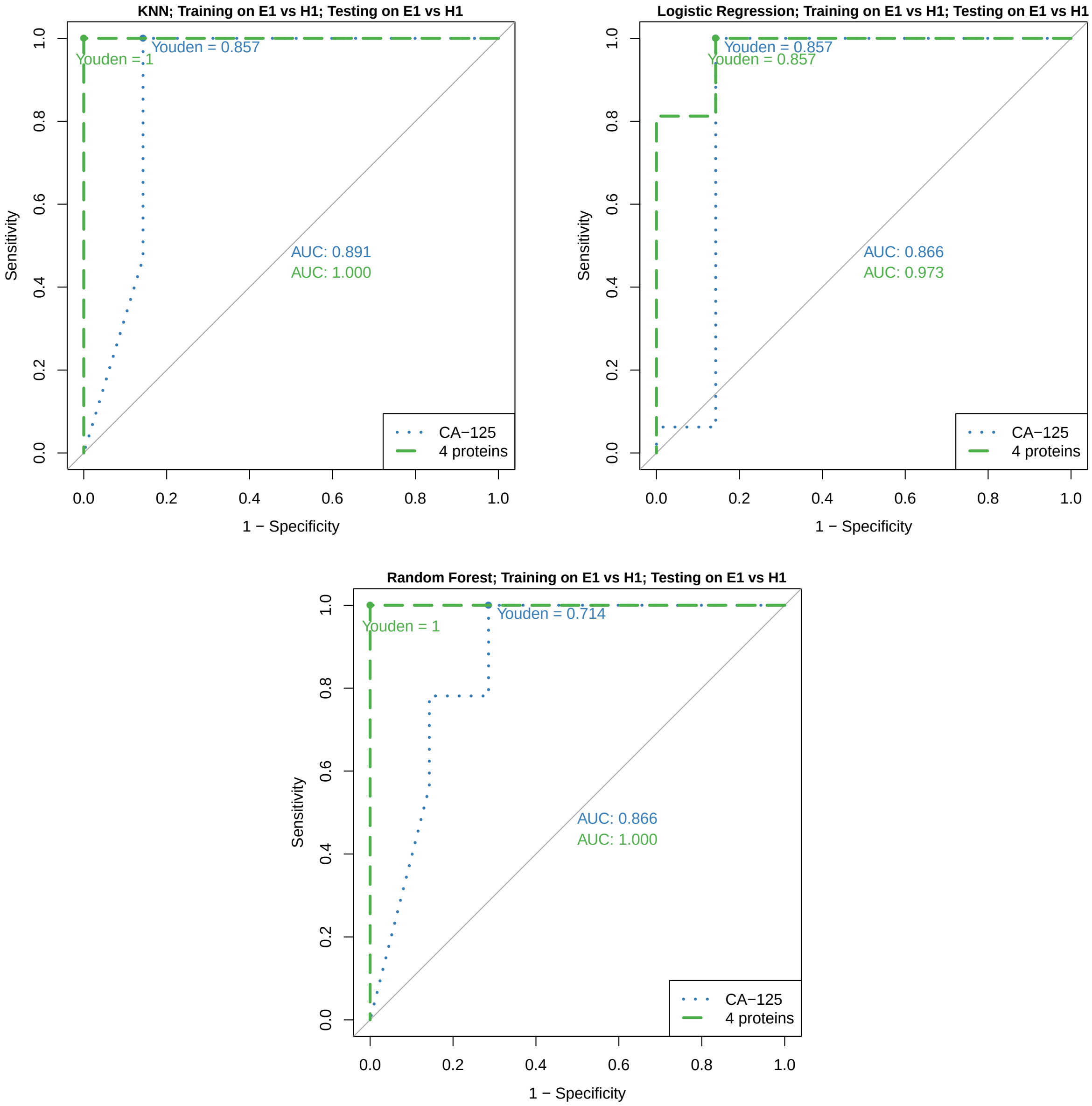

Figure S1: The AUC and Youden index when applying KNN, logistic regression, and random forest to identify control and healthy samples in experiment 1 in Table 8, using only CA-125 and all four proteins.

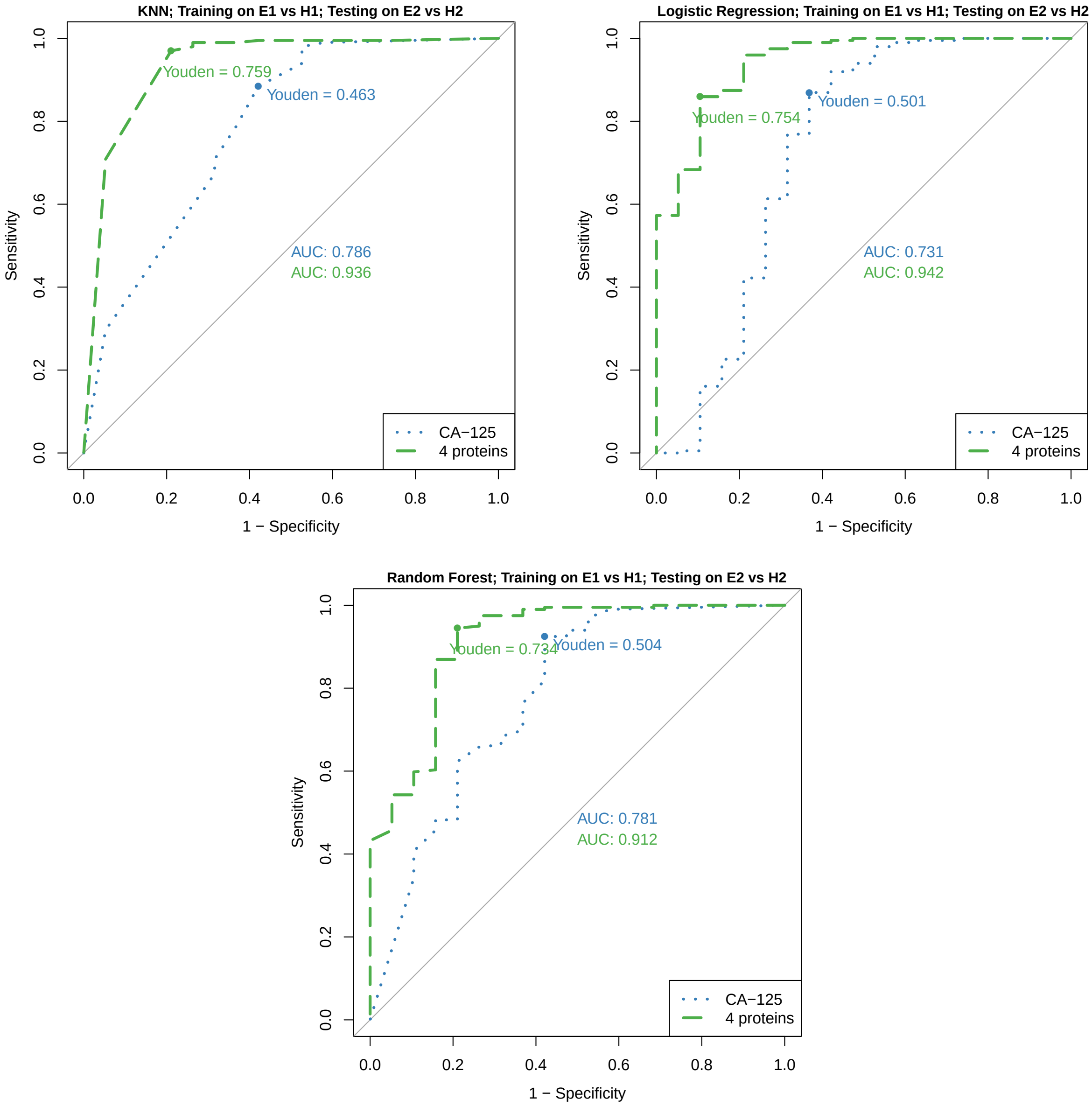

Figure S2: The AUC and Youden index when applying KNN, logistic regression, and random forest to identify control and healthy samples in experiment 2 in Table 8, using only CA-125 and all four proteins.

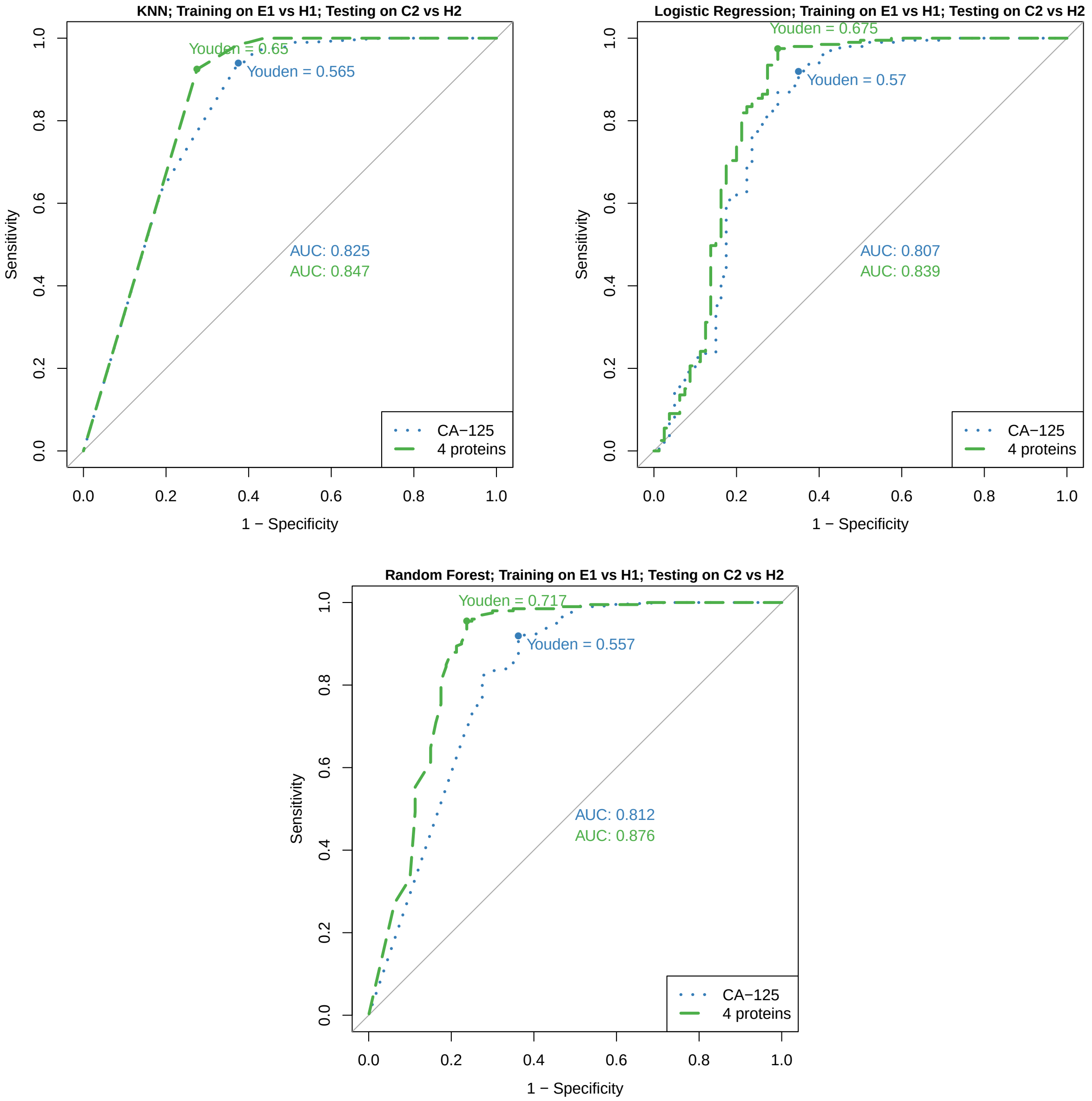

Figure S3: The AUC and Youden index when applying KNN, logistic regression, and random forest to identify control and healthy samples in experiment 3 in Table 8, using only CA-125 and all four proteins.

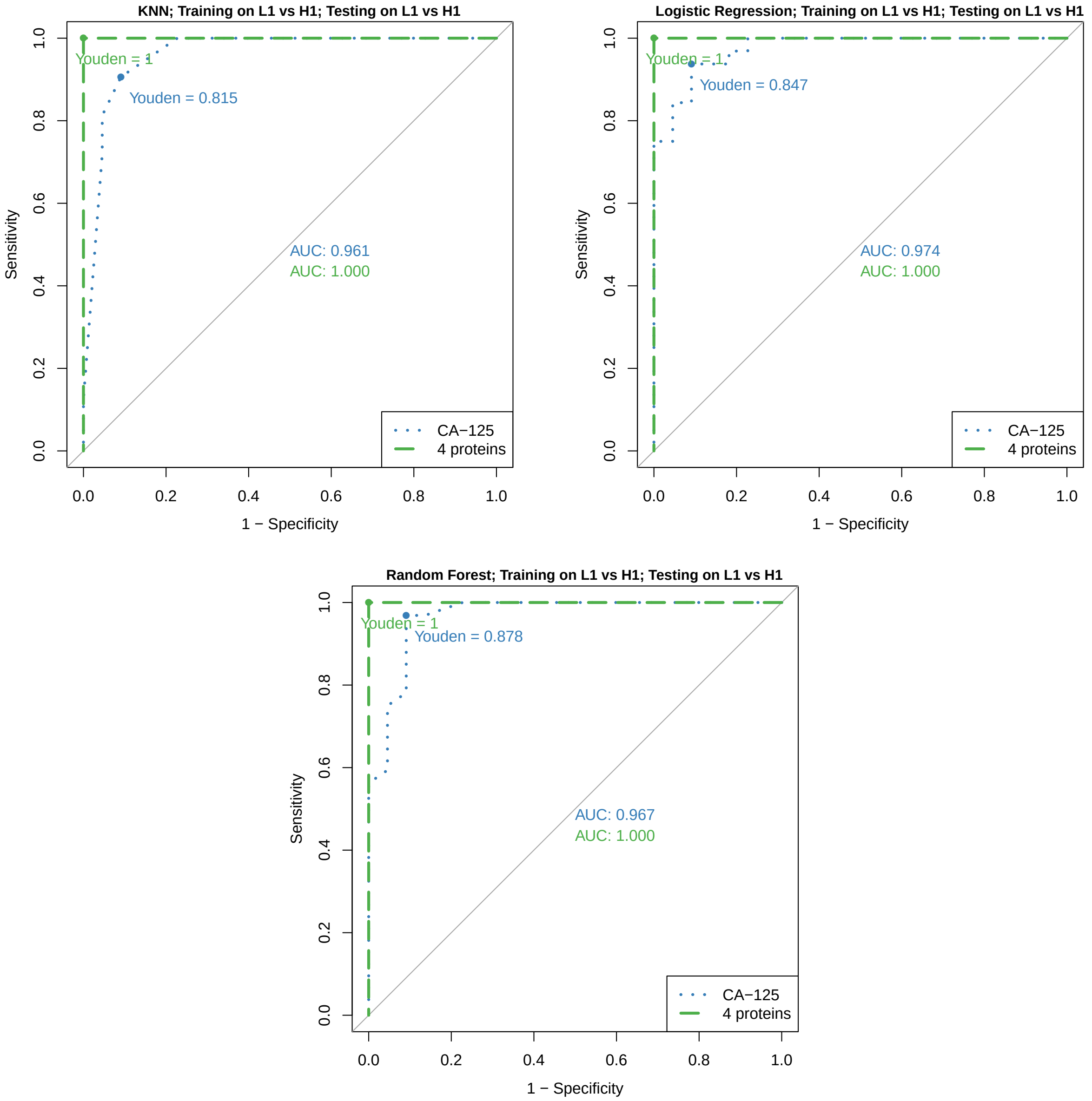

Figure S4: The AUC and Youden index when applying KNN, logistic regression, and random forest to identify control and healthy samples in experiment 4 in Table 8, using only CA-125 and all four proteins.

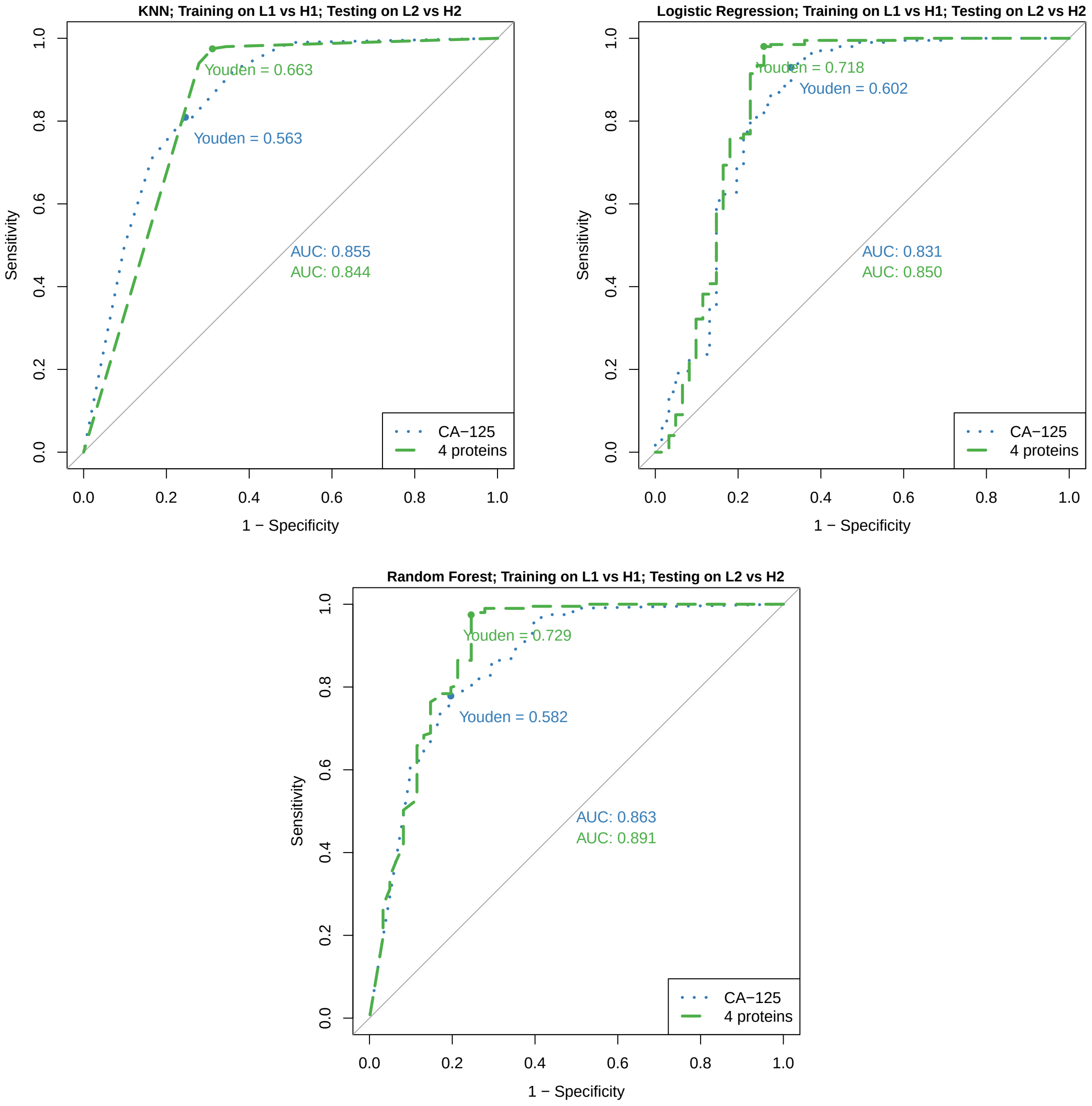

Figure S5: The AUC and Youden index when applying KNN, logistic regression, and random forest to identify control and healthy samples in experiment 5 in Table 8, using only CA-125 and all four proteins.

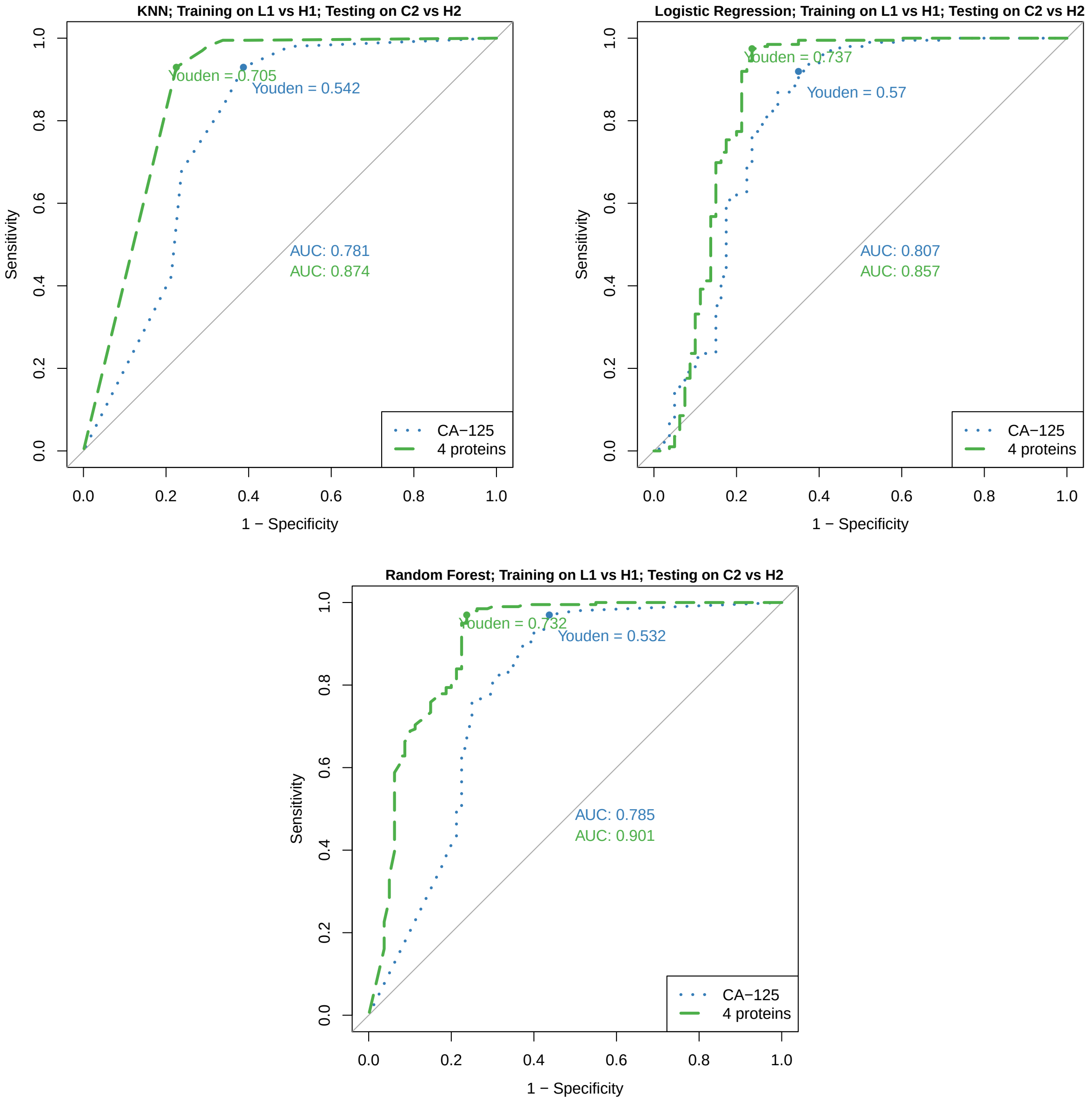

Figure S6: The AUC and Youden index when applying KNN, logistic regression, and random forest to identify control and healthy samples in experiment 6 in Table 8, using only CA-125 and all four proteins.

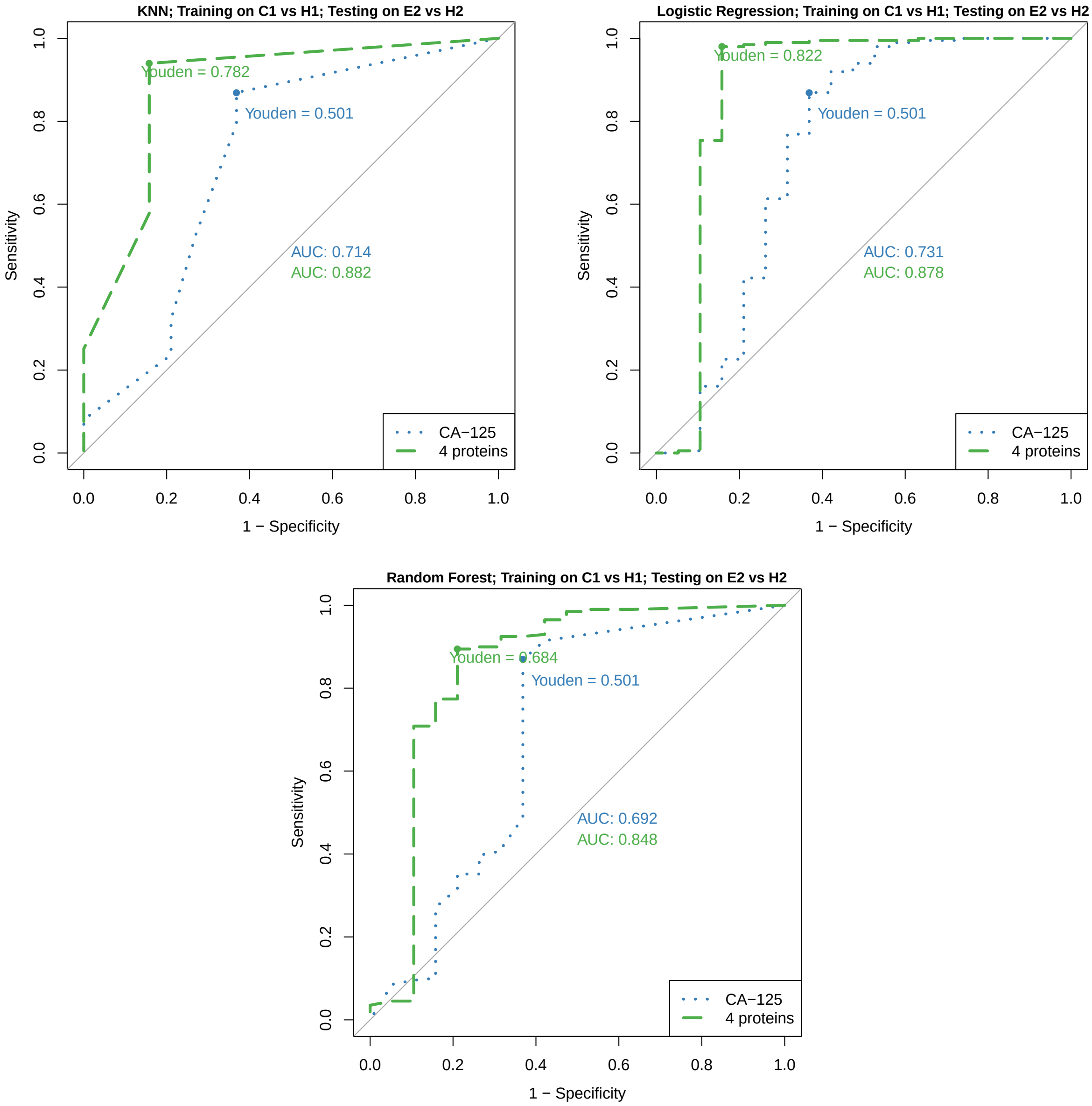

Figure S7: The AUC and Youden index when applying KNN, logistic regression, and random forest to identify control and healthy samples in experiment 7 in Table 8, using only CA-125 and all four proteins.

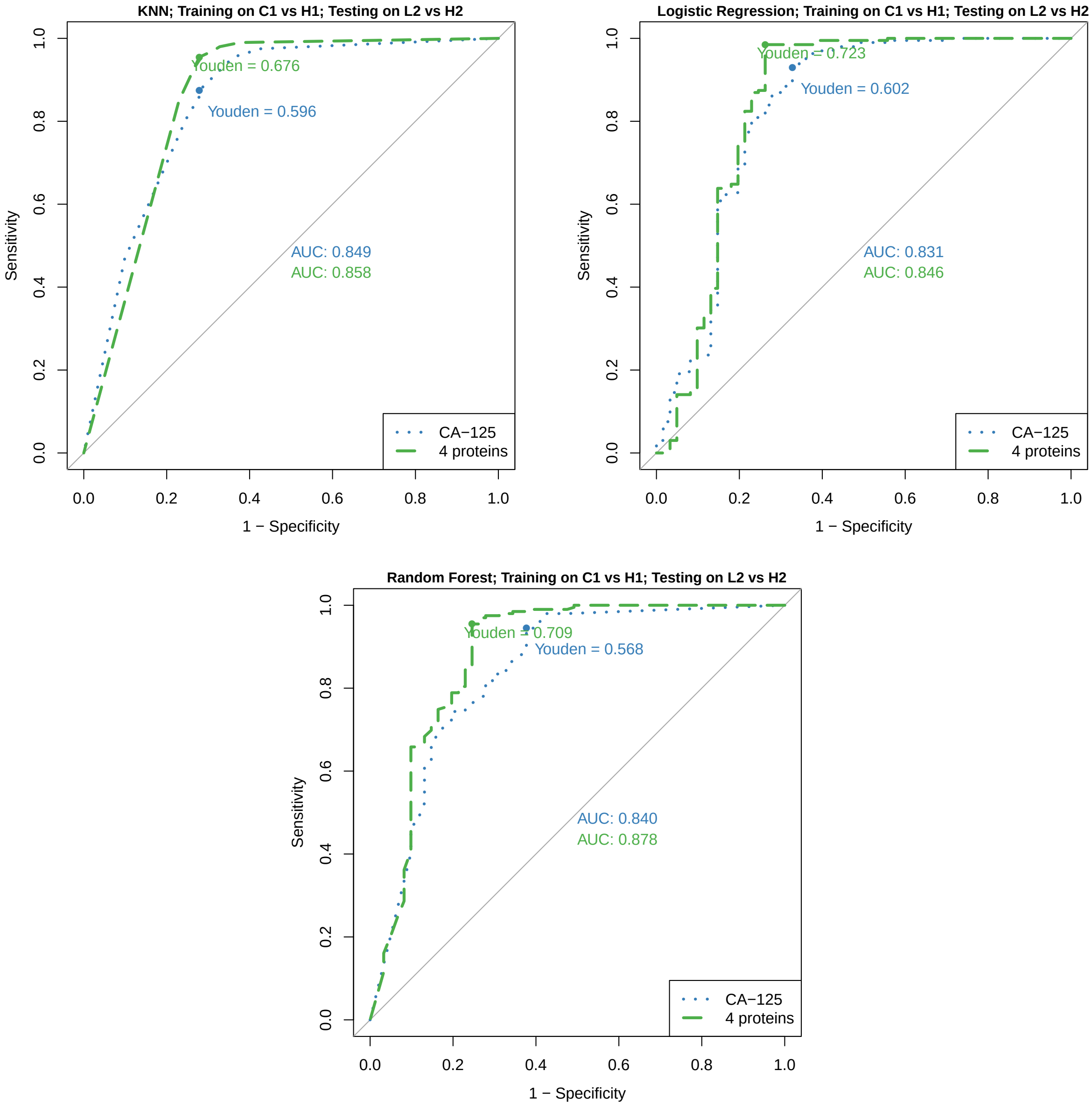

Figure S8: The AUC and Youden index when applying KNN, logistic regression, and random forest to identify control and healthy samples in experiment 8 in Table 8, using only CA-125 and all four proteins.

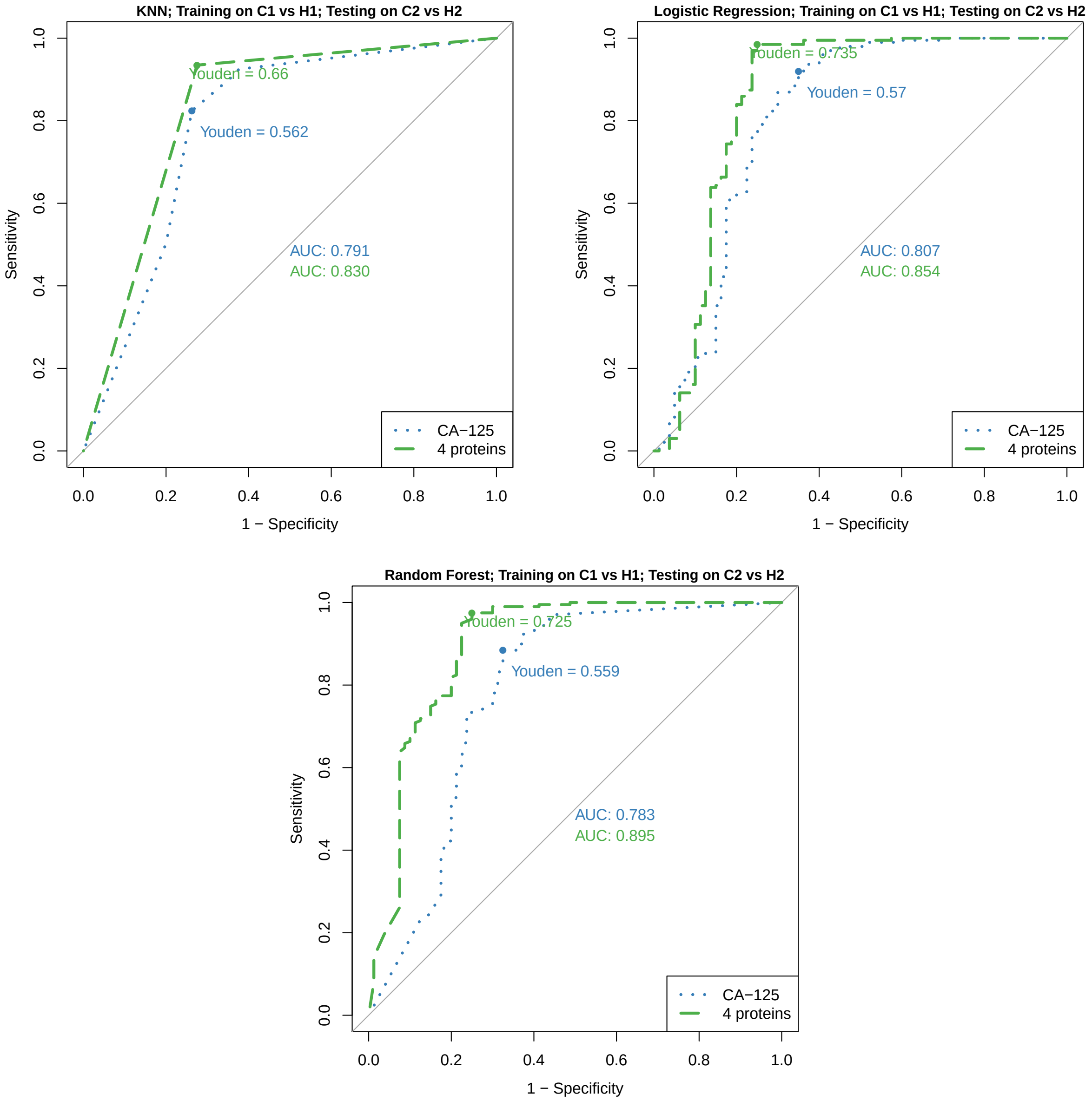

Figure S9: The AUC and Youden index when applying KNN, logistic regression, and random forest to identify control and healthy samples in experiment 9 in Table 8, using only CA-125 and all four proteins.

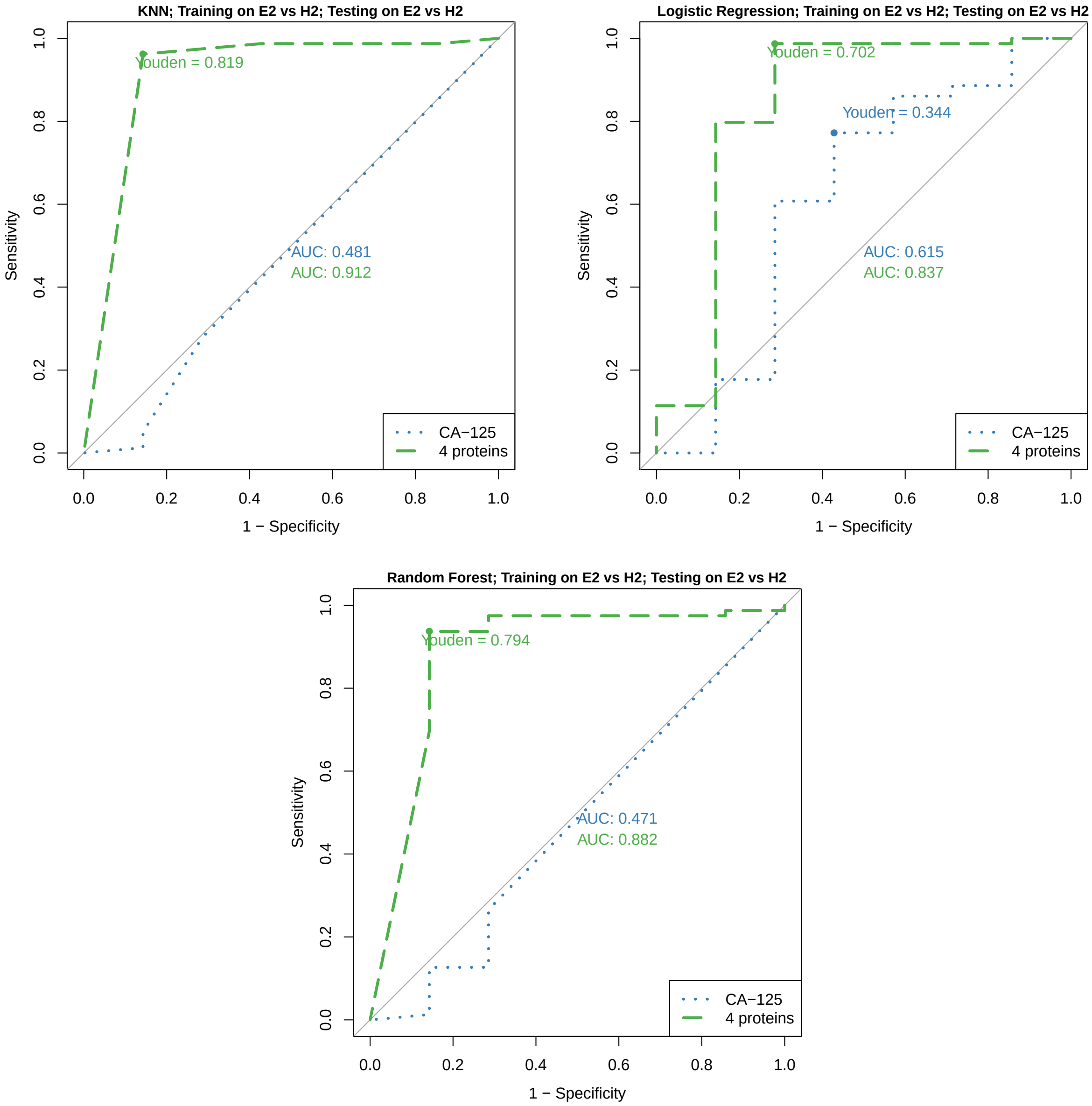

Figure S10: The AUC and Youden index when applying KNN, logistic regression, and random forest to identify control and healthy samples in experiment 10 in Table 8, using only CA-125 and all four proteins.

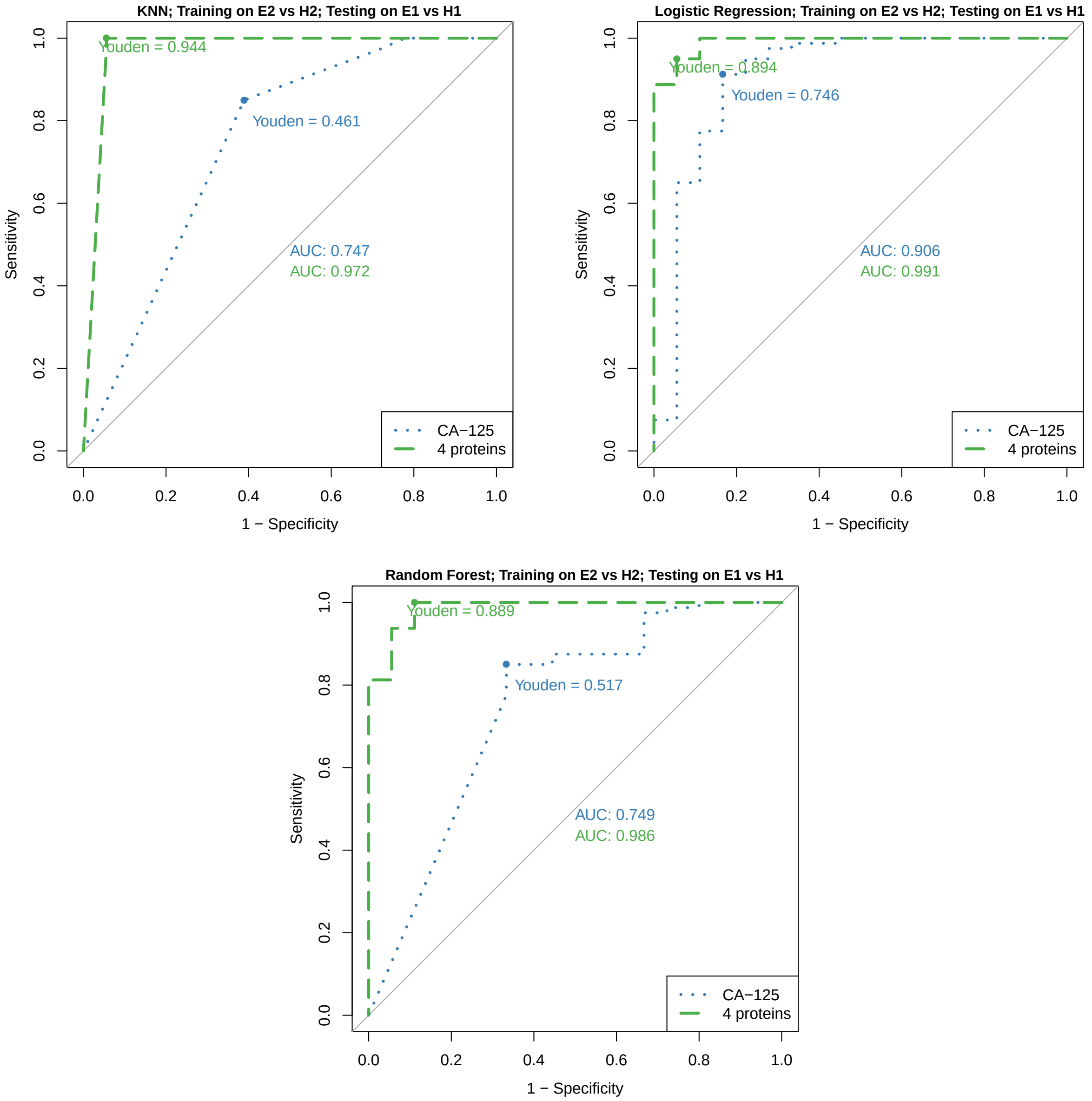

Figure S11: The AUC and Youden index when applying KNN, logistic regression, and random forest to identify control and healthy samples in experiment 11 in Table 8, using only CA-125 and all four proteins.

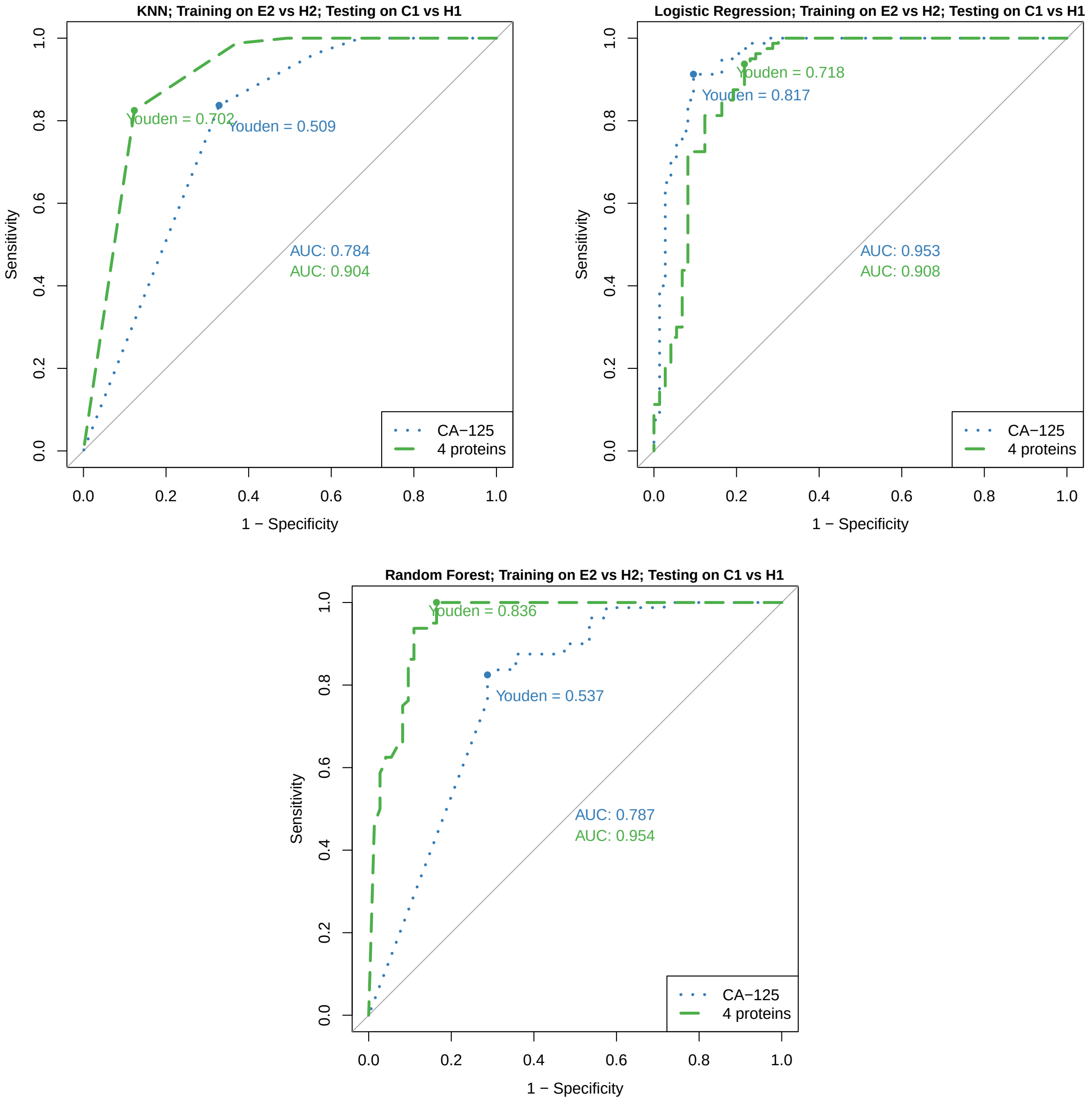

Figure S12: The AUC and Youden index when applying KNN, logistic regression, and random forest to identify control and healthy samples in experiment 12 in Table 8, using only CA-125 and all four proteins.

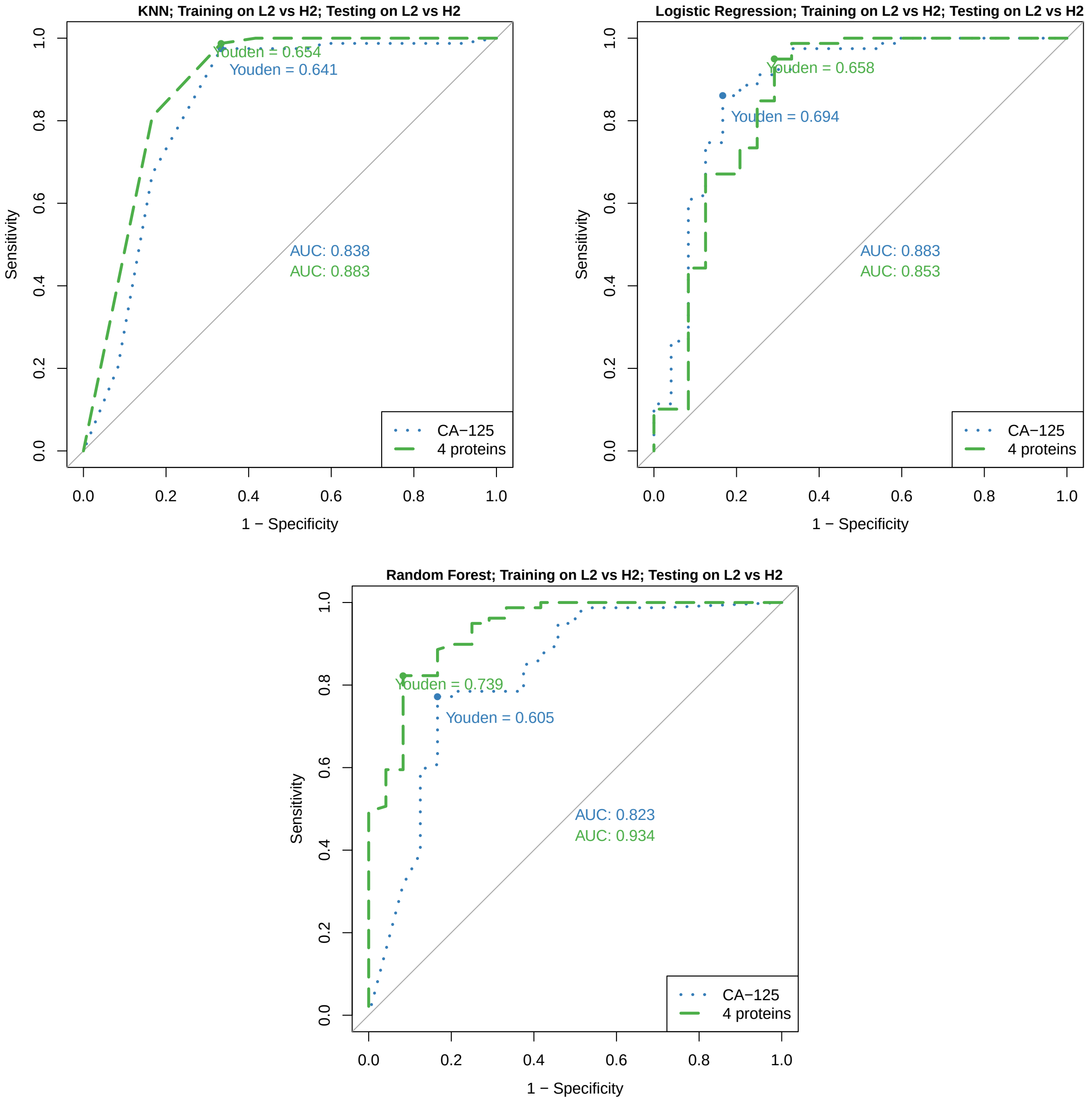

Figure S13: The AUC and Youden index when applying KNN, logistic regression, and random forest to identify control and healthy samples in experiment 13 in Table 8, using only CA-125 and all four proteins.

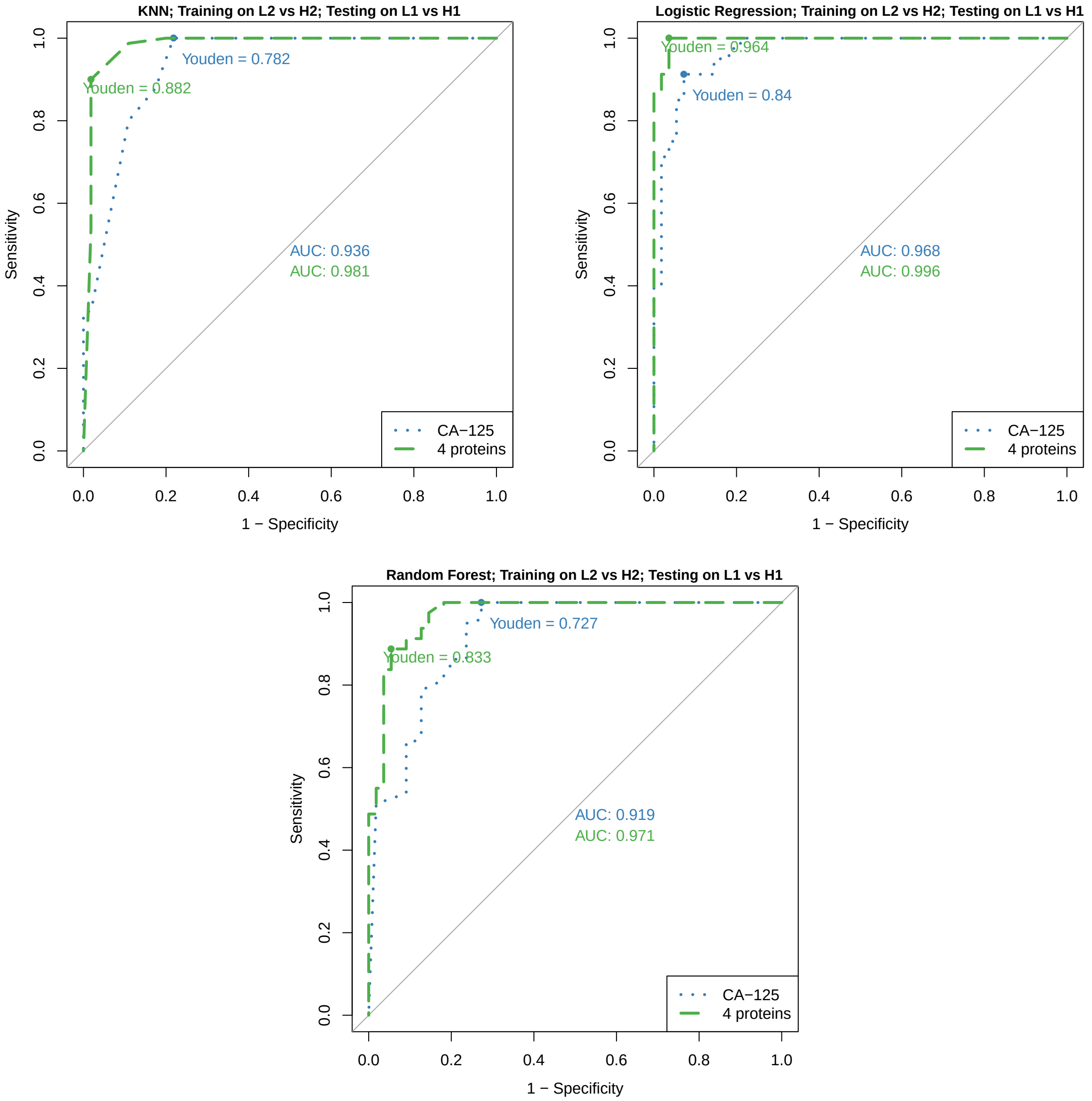

Figure S14: The AUC and Youden index when applying KNN, logistic regression, and random forest to identify control and healthy samples in experiment 14 in Table 8, using only CA-125 and all four proteins.

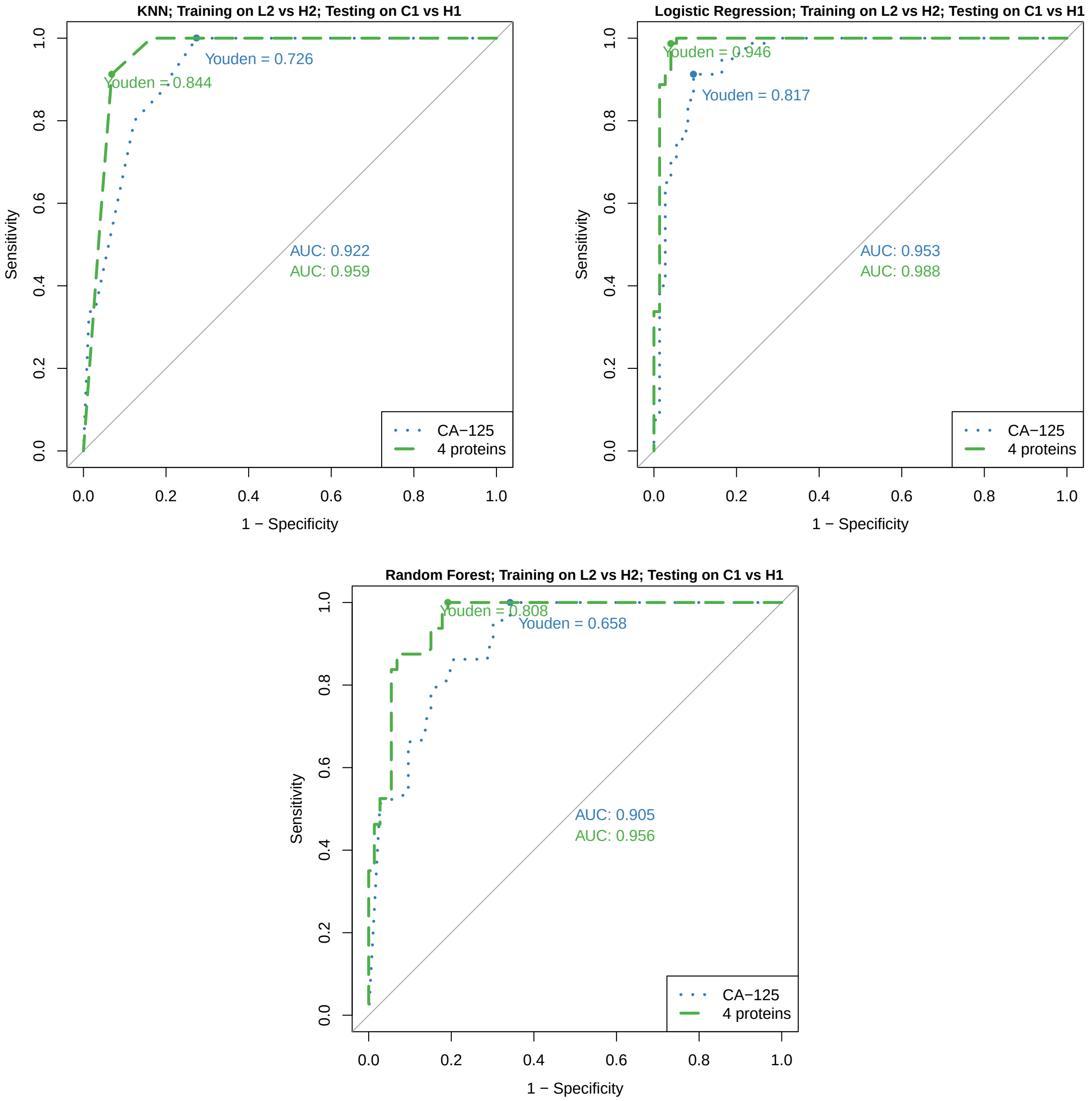

Figure S15: The AUC and Youden index when applying KNN, logistic regression, and random forest to identify control and healthy samples in experiment 15 in Table 8, using only CA-125 and all four proteins.

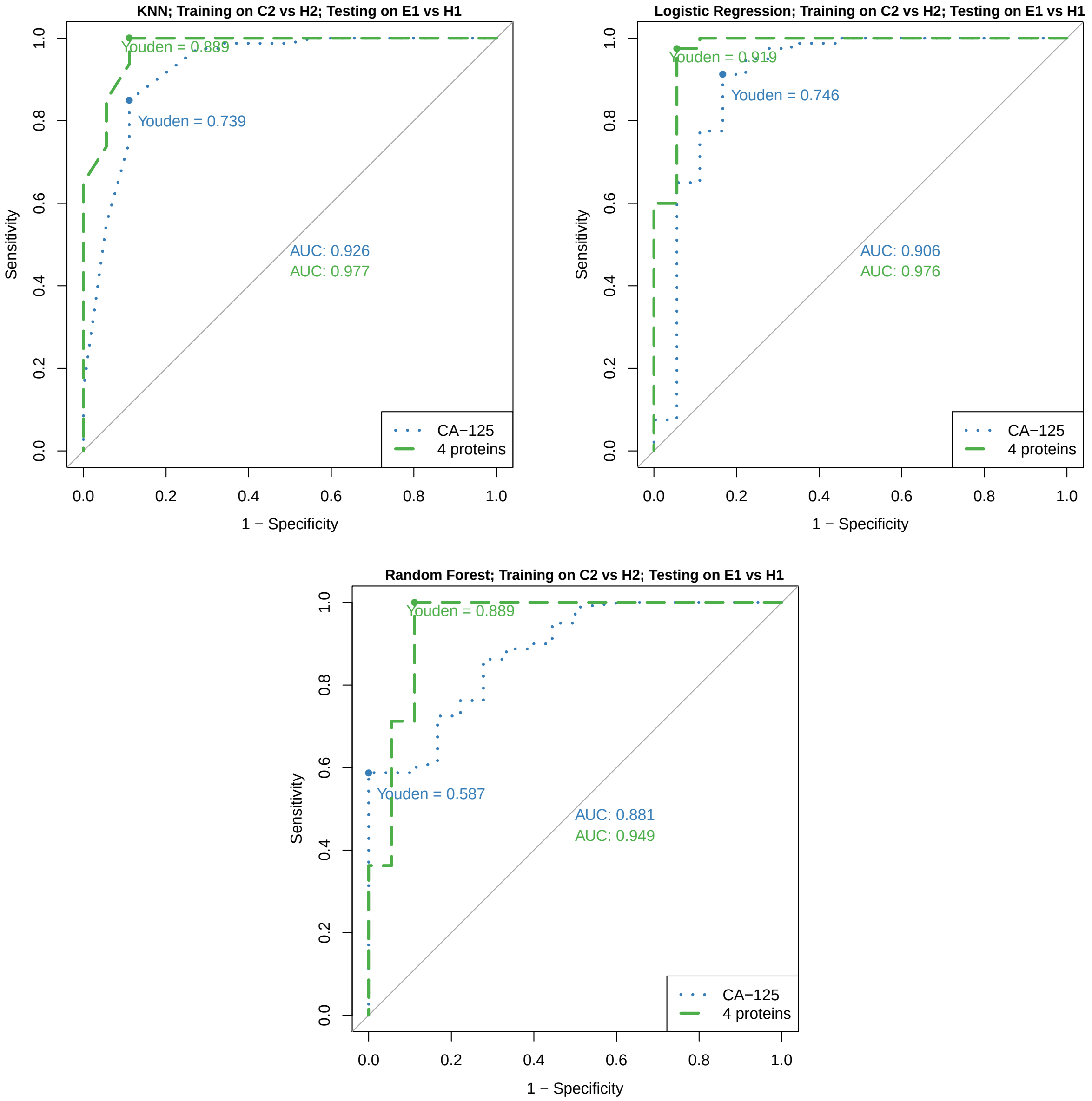

Figure S16: The AUC and Youden index when applying KNN, logistic regression, and random forest to identify control and healthy samples in experiment 16 in Table 8, using only CA-125 and all four proteins.

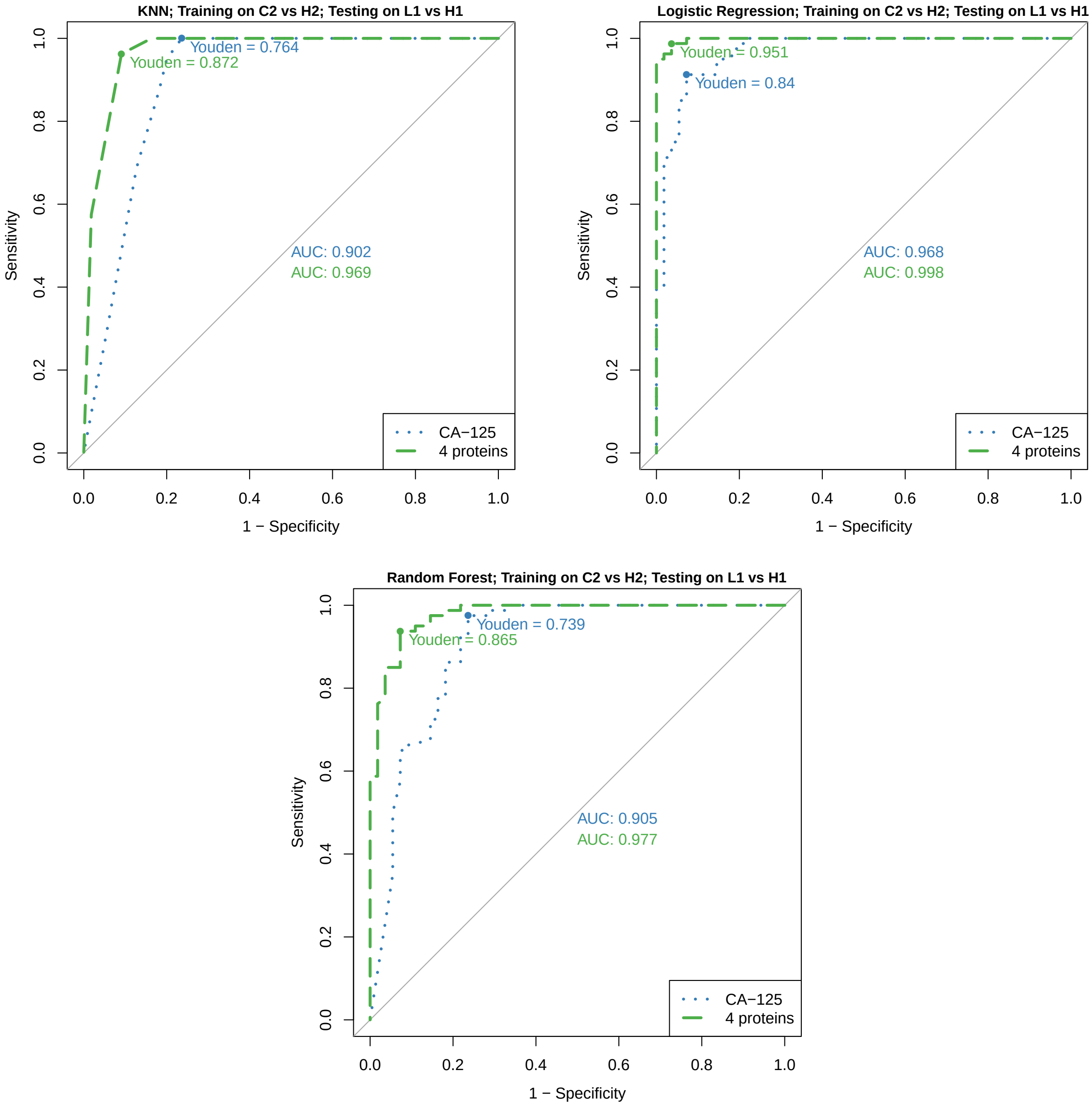

Figure S17: The AUC and Youden index when applying KNN, logistic regression, and random forest to identify control and healthy samples in experiment 17 in Table 8, using only CA-125 and all four proteins.

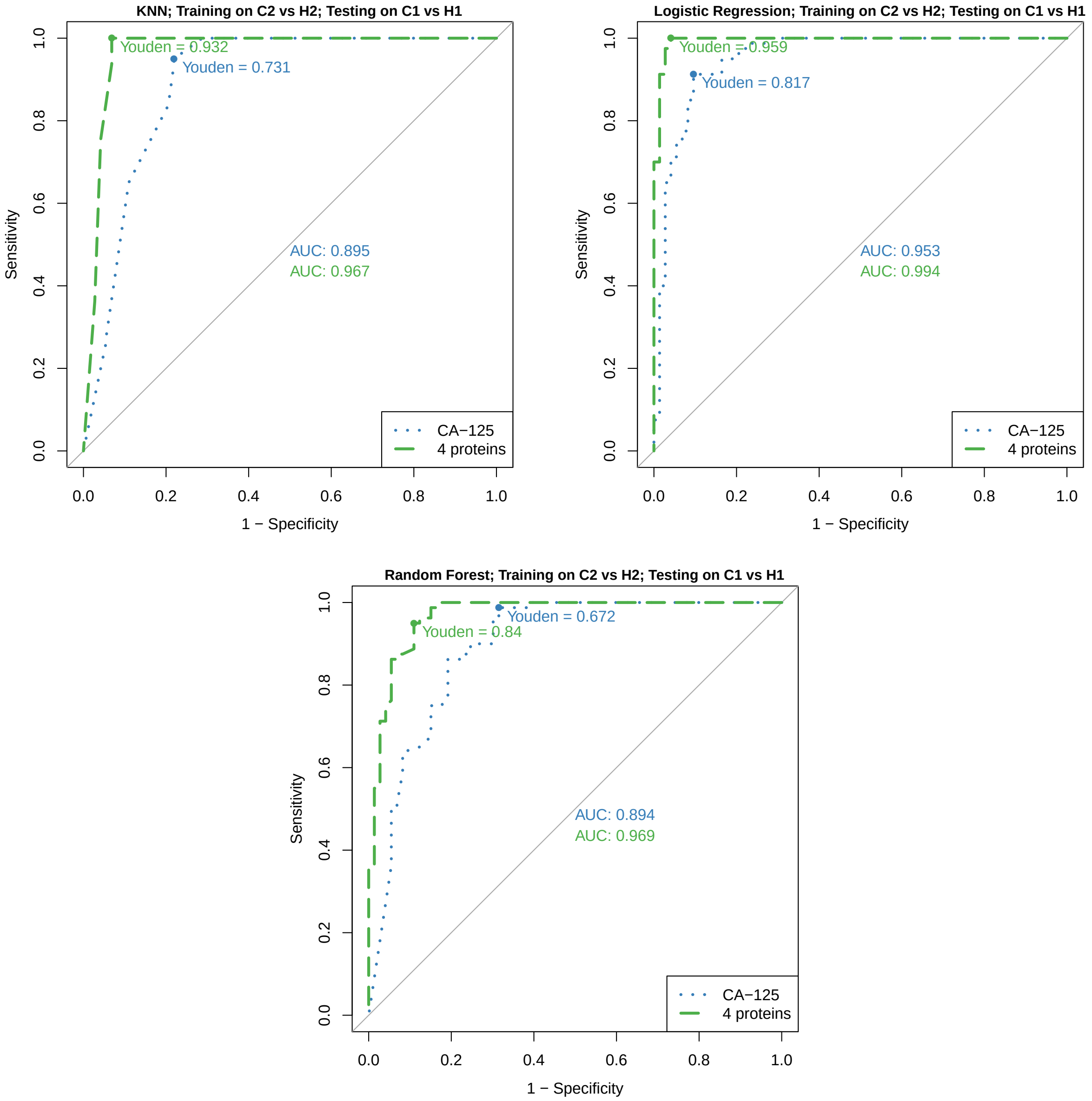

Figure S18: The AUC and Youden index when applying KNN, logistic regression, and random forest to identify control and healthy samples in experiment 17 in Table 8, using only CA-125 and all four proteins.
